# Liquid Chromatography Electrospray Ionization Tandem Mass Spectrometric (LC/ESI-MS/MS) Study for the Identification and Characterization of *In Vivo* Metabolites of Cisplatin in Rat Kidney Cancer Tissues: Online Hydrogen/Deuterium (H/D) Exchange Study

**DOI:** 10.1371/journal.pone.0134027

**Published:** 2015-08-05

**Authors:** Raju Bandu, Hyun Soo Ahn, Joon Won Lee, Yong Woo Kim, Seon Hee Choi, Hak Jin Kim, Kwang Pyo Kim

**Affiliations:** 1 Department of Applied Chemistry, College of Applied Sciences, Kyung Hee University, Yong-in City, Republic of Korea; 2 Department of Radiology, Pusan National University School of Medicine, Biomedical Research Institute, Pusan National University Hospital, Busan, Republic of Korea; 3 Department of Radiology, Pusan National University School of Medicine, Biomedical Research Institute, Pusan National University, Yangsan Hospital, Yangsan, Republic of Korea; The George Washington University, UNITED STATES

## Abstract

*In vivo* rat kidney tissue metabolites of an anticancer drug, cisplatin (cis-diamminedichloroplatinum [II]) (CP) which is used for the treatment of testicular, ovarian, bladder, cervical, esophageal, small cell lung, head and neck cancers, have been identified and characterized by using liquid chromatography positive ion electrospray ionization tandem mass spectrometry (LC/ESI-MS/MS) in combination with on line hydrogen/deuterium exchange (HDX) experiments. To identify *in vivo* metabolites, kidney tissues were collected after intravenous administration of CP to adult male Sprague-Dawley rats (n = 3 per group). The tissue samples were homogenized and extracted using newly optimized metabolite extraction procedure which involves liquid extraction with phosphate buffer containing ethyl acetate and protein precipitation with mixed solvents of methanol-water-chloroform followed by solid-phase clean-up procedure on Oasis HLB 3cc cartridges and then subjected to LC/ESI-HRMS analysis. A total of thirty one unknown *in vivo* metabolites have been identified and the structures of metabolites were elucidated using LC-MS/MS experiments combined with accurate mass measurements. Online HDX experiments have been used to further support the structural characterization of metabolites. The results showed that CP undergoes a series of ligand exchange biotransformation reactions with water and other nucleophiles like thio groups of methionine, cysteine, acetylcysteine, glutathione and thioether. This is the first research approach focused on the structure elucidation of biotransformation products of CP in rats, and the identification of metabolites provides essential information for further pharmacological and clinical studies of CP, and may also be useful to develop various effective new anticancer agents.

## Introduction

Identification of metabolites is crucial in the drug discovery and development process to optimize lead compounds for further development. The information generated in the early discovery phase of metabolic identification can be used to identify lead compounds and undesirable metabolic products followed by optimization of pharmacokinetic and safety profiles. Identification of reactive or toxic metabolites is essential to avoid the toxicity and helps to modify the structure by means of chemical transformations. These studies are usually carried out by employing in *vitro* and *in vivo* systems followed by using modern analytical instruments. Liquid chromatography tandem mass spectrometry (LC-MS/MS, MS^*n*^) combined with online H/D exchange (HDX) experiments and accurate mass measurements is the most popular analytical technique for metabolite identification [1–13]. Accurate and reproducible identification of metabolites in tissue homogenate samples is of high importance to characterize animal models and to identify metabolic changes that occur in different tissue types in specific diseases. However, the extraction of metabolites from tissue homogenates is one of the most labor intensive steps for metabolomic studies to yield reproducible results between repeated samples.

Cisplatin (cis-diamminedichloroplatinum(II)) (CP) is the most potent chemotherapeutic antitumor drug used in the treatment of testicular, ovarian, bladder, cervical, esophageal, small cell lung, head and neck cancers [14,15]. The mechanistic pathway of CP involves the formation of adducts with DNA nucleobases by covalent interactions which result in cross links between adjacent nucleobases that block DNA replication, transcription and ultimately cell division [16]. The hydrolysis of CP yields the monohydrated CP complex (cis-diammineaquachloroplatinum (II); MHC) which is most important for anticancer activities as well as side effects of CP [17, 18]. Numerous side effects are related to CP therapy like ototoxicity, gastrointestinal toxicity, tubular necrosis, oxidative stress, inflammation, apoptosis, myelosuppression, neurotoxicity, nephrotoxicity (kidney injury) and nausea [19–22]. The clinical use of CP is often more complicated by dose-limiting major side effect nephrotoxicity [23]; and the mechanism of nephrotoxicity remains obscure. Even though the mechanism of nephrotoxicity remains unclear, kidney CP levels have been shown to be several fold higher than plasma levels which were found in other organs following administration of CP to animals [24, 25]. It was reported earlier that kidney CP concentrations are higher in patients with evidence of nephrotoxicity than patients without kidney damage [26]. After intravenous administration of CP, it reacts extensively with proteins, nucleotides and low molecular mass compounds to form fixed and mobile metabolites, respectively. Furthermore, some unchanged CP also present in the initial distribution phase after intravenous administration of CP [27]. Moreover, unchanged CP and several low molecular mass metabolites (mobile metabolites) are actively secreted and/or reabsorbed by the kidney during early phase [27–30]. It has been shown that protein bound CP (fixed metabolites) does not cause the nephrotoxicity in rats [31]. Kidneys represent the main route of CP and its metabolites excretion with proximal tubule cells and tissues which are the primary sites of CP accumulation [32]. Currently, there is a great deal of interest in the analysis of CP and its metabolites, since it increases the cure rates dramatically for various types of human cancers in the chemotherapy [33]. However, the dose of CP is limited because of its major toxic side effects such as nephrotoxicity and severe nausea. The nephrotoxicity of CP may be directly or indirectly linked to specific metabolites [34]. To understand the relationship between toxic effects and CP disposition, it is essential to demonstrate the distribution and biotransformation of CP in the organs showing side effects. Several studies have been reported on HPLC analysis [35–42] and pharmacokinetic studies of CP in animals and humans [21, 43–45]; but most of them are in plasma and/or urine. EI-Khateeb et al., [46] have investigated a study on the optimized conditions for HPLC separation of CP and its two hydrolyzed products. Ehrsson et al., have demonstrated the separation and identification of CP, transplatin and their hydrated complexes using porous graphitic carbon and electrospray ionization mass spectrometry (ESI-MS) [47]. Recently, Du et al., have investigated a study on the hydrolysed products of CP in various buffer conditions and the effect of the buffer on hydrolysis of CP by using Fourier transform ion cyclotron resonance mass spectrometry (FTICR-MS) [48]. Some researchers have demonstrated that the production of metabolites is a necessary prerequisite for the nephrotoxicity in the chemotherapy of CP [49–51]. Several studies have been under taken to understand the cause of nephrotoxicity and to correct the problems [52–54]. However, none of these studies provided essential information on the intact CP and its metabolites. It was reported earlier that chlorine atoms from CP can be readily replaced by water and other nucleophiles resulting the formation of platinum complexes [55–57]. Until recent investigations into the mechanism and extent of metabolism of CP were limited by the lack of a suitable LC-MS method and metabolite identification. Thus, it is necessary to develop the LC-MS/MS method for the identification and characterization of metabolites of CP which will become very important for the elucidation of its mode of action, toxicity and optimization of the chemotherapy.

In the present study, we have developed and optimized the LC-MS/MS method for the identification and characterization of unknown *in vivo* metabolites of CP after intravenous administration of drug to adult male Sprague-Dawley rats by employing following steps. (i) Removal of whole kidney from rats (ii) Homogenization of kidney tissues (iii) Extraction of CP and its metabolites from kidney tissue homogenate samples using newly optimized metabolite extraction procedure (iv) LC/ESI-HR-MS analysis of extracted tissue homogenate sample solutions containing drug and its metabolites and (v) Structural characterization of CP and its metabolites using LC-MS/MS experiments combined accurate mass measurements in conjunction with online HDX experiments.

## Experimental

### Chemicals and reagents

High pure CP was a gift sample from JW Pharmaceuticals, Seoul, Republic of Korea. HPLC grade methanol, chloroform, water and analytical reagent grade formic acid, ethyl acetate, triethylamine, phosphate buffer, deuterium oxide (D_2_O, 100% atom ‘D’), deuterated methanol and _D_-formic acid (98% atom ‘D’) used in the present study were purchased from Sigma Aldrich, Seoul, Republic of Korea and used without further purification.

### Animal experiments

Animals were supplied by SAMTACO BIOKOREA, Osan, Republic of Korea. All animal experiments were carried out according to the guidelines by Institutional Animal Care and Use Committee (IACUC), Pusan National University, Busan, Republic of Korea. The study was approved by the IACUC (Approval No: 2014–062), Pusan National University, Busan, Republic of Korea. The animal house is maintained at temperature 22 ± 2°C with relative humidity of 50 ± 15% and 12 h dark/light cycle. Animals were kept in an environmentally controlled breeding room with standard laboratory food and water for three days prior to the experiments. The animals were fasted for 12 h with free access to water prior to the drug administration. Three sets (n = 3 per group for biological replicates) of adult male Sprague-Dawley rats (290–310g) with similar weight and age were utilized to carry out the present study for unique results and to avoid the variation in the total number of metabolites. Another set of rats (n = 3) with same weight and age was used as control specimens. Each rat from three sets were intravenously administered with 3mg/kg dose of CP and all the rats were sacrificed by decapitation method at different time points, 2h, 6h, 12h, 24h and 36h after dosing. All the animals were monitored carefully for every hour after the administration of drug. Whole kidney was excised, washed with normal saline, blotted dry with filter paper and then weighed accurately. The homogenization and extraction procedure of tissues has been explained below in the context of the sample preparation section.

### Instrumentation

#### LC-MS/MS equipment and conditions

The HPLC analysis was performed on an Agilent 1290 infinity series HPLC instrument (Agilent Technologies, USA) equipped with a binary pump (G4220A, USA), a variable wavelength detector (VWD) (G1314F, USA), an auto sampler (G4226A, USA), a thermostat (G1330B, USA), and a column compartment (G1316C, USA). Effective chromatographic separation was achieved on a Agilent ZORBAX SB C-18 column (2.1 X 50 mm, 1.8 μm) using the mobile phase consisting of 0.1% formic acid in water (Solvent A) and methanol (Solvent B) in a gradient elution mode. The optimized gradient program was set as follows: (T_min_ / % solution of B): _0_/15, _6_/30, _12_/65, _20_/65, _30_/90, _35_/15. The flow rate of the mobile phase was 0.4 mL/min, the column temperature was at 30°C and the injection volume was 5μL.

LC-MS analysis was performed on a quadrupole time-of-flight (Q-TOF) mass spectrometer (Q-TOF LC/MS 6550 series classic G6550A, Agilent Technologies, USA) equipped with an ESI source. The data acquisition was under the control of Mass Hunter workstation software. The typical operating source conditions for MS scan in positive ion ESI mode were optimized as follows; the fragmentor voltage was set at, 175 V; the capillary at, 3000–3500 V; sheath gas temp at, 350°C; sheath gas flow at, 11 L/min; nitrogen was used as the drying (250°C; 13 L/min) and nebulizing (45 psi) gas. For full scan MS mode, the mass range was set at *m/z* 100–3000. For collision-induced dissociation (CID) experiments, keeping MS^1^ static, the precursor ion of interest was selected using the quadrupole analyzer and the product ions were analyzed using a time-of-flight (TOF) analyzer. Ultra high pure nitrogen was used as collision gas. All the spectra were recorded under identical experimental conditions and are average of 20–25 scans. The elemental compositions from the accurate mass measurements of *m*/*z* values and data processing of total ion chromatograms (TICs) were also carried out by using Mass Hunter workstation software. The trace level metabolites were verified by extracting the masses of protonated metabolites using extracted ion chromatograms (EICs) after the post run analysis. The metabolites were obtained from the metabolic pathways in Agilent Metabolite ID (Agilent Technologies). The elemental compositions of protonated metabolites and proposed MS/MS fragment ions were confirmed by accurate mass measurements.

For online HDX LC-MS experiments, deuterated water was used to prepare the mobile phase *i*.*e*. deuterium oxide (D_2_O, 100% atom ‘D’) as Solvent A with 0.1% _D_-formic acid (98% atom ‘D’) and deuterated methanol as solvent B.

### Sample Preparation

In this study, liquid extraction with phosphate buffer containing ethyl acetate and protein precipitation (PPT) with mixed solvents of methanol-water-chloroform followed by solid-phase clean-up procedure on Oasis HLB 3cc cartridges was used for the extraction of CP and its metabolites from kidney tissue homogenates. The following steps are involved in the process of extraction of metabolites from tissue homogenates. The frozen tissues of kidney were taken out from -80°C storage, washed with normal saline, blotted dry with filter paper, finely diced with scissors and weighed accurately after fat and connective tissue had been trimmed away. A 100mg of dissected tissue was placed into precooled (dry ice) 2ml homogenization tubes containing ceramic beads (1.4 mm diameter). The pre-cooled extraction solvents were used for the extraction of metabolites from homogenized tissues. A total of 1mL solvent volume was used for 100 mg of tissue. The homogenized tissue sample was transferred into a cleaned poly-propylene centrifuge tube and added 600 μL of phosphate buffer (10mM) followed by 400μL of ethyl acetate. The mixture was vortexed for 2 min, sonicated for 5 min (the tube was refrigerated in an ice-bath) and centrifuged at 13,000 rpm for 10 min at 4°C. Both aqueous and organic layers were carefully collected into separate polypropylene tubes. For more precipitation of proteins from the collected supernatant (200 μL), 400 μL of methanol, 100 μL of water followed by 300 uL of chloroform were added and vortexed the mixture for 1 min, and then centrifuged for 10 min at 4000 rpm at 4°C. A 200 μL of supernatant was collected into another cleaned poly-propylene centrifuge tube and added 400μL each of chloroform and water. A biphasic mixture was formed after vortexed for 1 min and centrifuged for 10 min at 4000 rpm. The upper polar (water) and lower nonpolar (chloroform) layers were carefully collected. The lower nonpolar layer was frozen and upper polar layer was collected into another tube and then it is subjected to solid phase extraction (SPE) using Oasis HLB 3cc cartridges. Simultaneously, the initial aqueous layer which was collected in the first stage (collected from ethyl acetate/phosphate buffer) was also subjected to SPE. The SPE column was conditioned by washing with 1 ml each of methanol-triethylamine (93:7, v/v), water and phosphate buffer (5mM; pH 7.4). After that, the sample was loaded to the column, washed with 1 mL each of water and methanol-water (50:50, v/v), and the analytes were eluted with methanol-triethylamine (93:7, v/v). As the main aim of our study is focused on the identification of total *in vivo* metabolites of CP, the aliquots from both SPE’s were pooled together and they were finally pooled with the acetonitrile layer. Further, the samples were filtered and evaporated to dryness under a gentle stream of nitrogen at 45°C. After that, the residue was reconstituted in 100 μL methanol-water (50:50, v/v) and only 5 μL aliquots were injected into LC-MS system. The extracted tissue homogenates were stored at -20°C before and after analysis.

### Optimization of the Extraction Method

The extraction of analytes from solid biological matrices like tissues require more vigorous approaches than those used for biological fluids such as urine and plasma. Thus, the homogenization is a critical step in the method development for separation and quantitative determination of drug in tissues, because it greatly affects the extraction recovery and optimization of analytical method. The majority of the previous researchers have used the PPT method for the extraction of CP from the biological matrices. For PPT, they used different solvents like hot water [39], methanol [42], acetonitrile [45] and the mixture of acetonitrile/dichloromethane [45] for the extraction of CP from the biological matrices. Some researchers have used sucrose containing phosphate buffer for the extraction of CP from tissues [27].

In our study, for the extraction of CP and its metabolites from kidney tissues, initially we tried PPT and liquid-liquid extraction (LLE) methods with various solvents like methanol, chloroform, methanol-water, chloroform-water, methanol-water-chloroform, phosphate buffer (10mM), methanol/phosphate buffer, ethanol/phosphate buffer, water/ethyl acetate to check the potential differences and to remove the biological interferences, and for better extraction of analytes from tissues. We also tried individual SPE method using Oasis HLB 3cc cartridges for the extraction of CP with different solvents. From these methods, we observed that no single solvent and single method worked best for the extraction of all the metabolites from kidney tissues without showing any significant matrix interference. Furthermore, not these tissues behaved similarly with regard to different extraction solvents in different extraction methods (PPT, LLE and SPE) that were tried. After several trials, liquid extraction with phosphate buffer containing ethyl acetate and protein precipitation with mixed solvents of methanol-water-chloroform followed by solid-phase clean-up procedure on Oasis HLB 3cc cartridges was found to be suitable for the extraction of CP and its metabolites from kidney tissue homogenates since this extraction method was able to detect the more number of metabolites with cleaner chromatogram without showing any significant biological interferences. Moreover, the protocol that we developed here could detect even very low level metabolites with good recovery which was not possible with the individual PPT, LLE and SPE methods that were used for the extraction of metabolites of CP.

The reproducibility of both homogenization and extraction protocol was tested in three independent experiments using kidney tissue samples of the same rat as well as different rats from each group. Each tissue homogenate was analyzed three times to obtain the reproducible results and to avoid the variation in the total number of metabolites. It can be noted that the results obtained from independent experiments are very similar to each other.

## Results and Discussion

CP undergoes metabolic activation in the kidney to more potent toxic materials. This process initiates with the formation of glutathione conjugates in the circulation which may presumably mediated by glutathione-S-transferase [58, 59]. As the glutathione-conjugates pass through the kidney, they cleaved to cysteinyl-glycine-conjugates by gamma glutamyl transpeptidase (GGT) expressed on the surface of the proximal tubule cells [60, 61]. The cysteinyl-glycine-conjugates are further metabolized to cysteine-conjugates by aminodipeptidases, also expressed on the surface of the proximal tubule cells [60]. The cysteine-conjugates are transported into the proximal tubule cells, where they are further metabolized by cysteine-S-conjugate beta-lyase to highly reactive thiols [60–62]. The dissociation of one of the chlorine atoms from CP results in a positive charge on the platinum that will attract the negatively charged sulfur present on the active thio group containing molecules like methionine, cysteine, acetylcysteine, glutathione and thioether to form various toxic metabolites.

**Fig 1:**
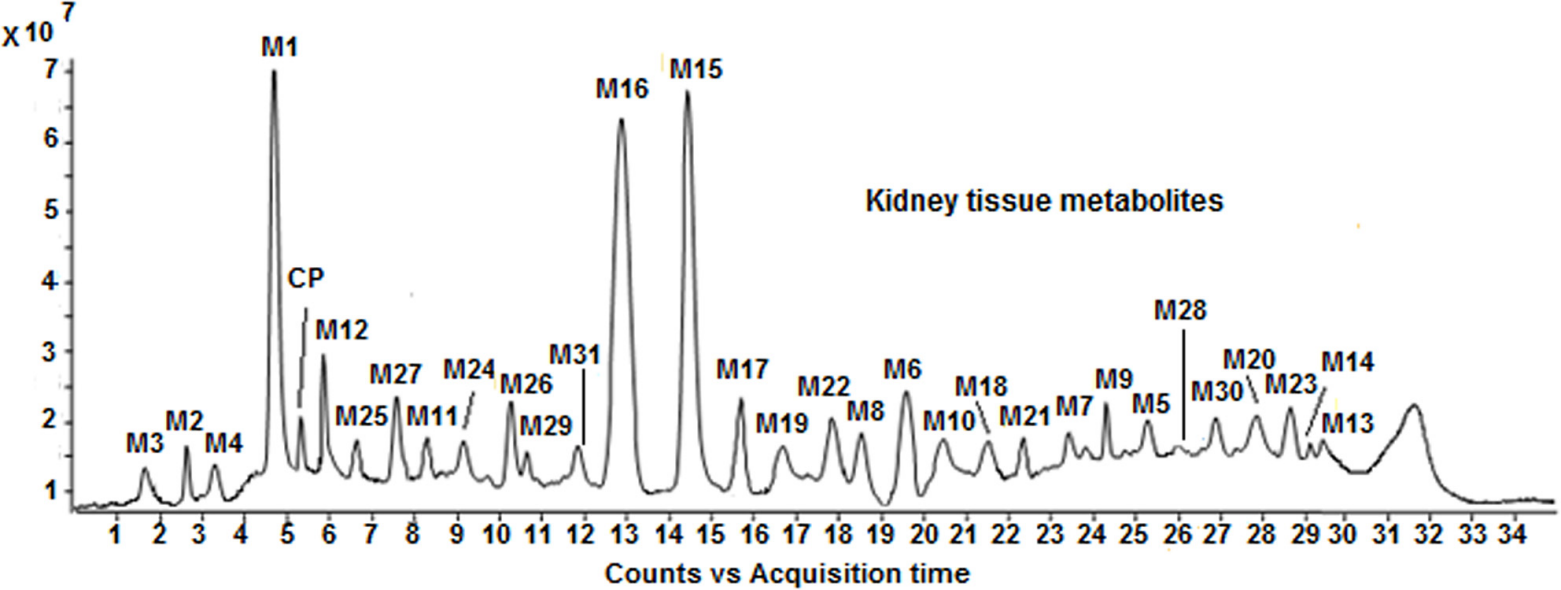
LC/ESI-MS-TIC of metabolites of CP formed in kidney tissue homogenate sample.

**Fig 2:**
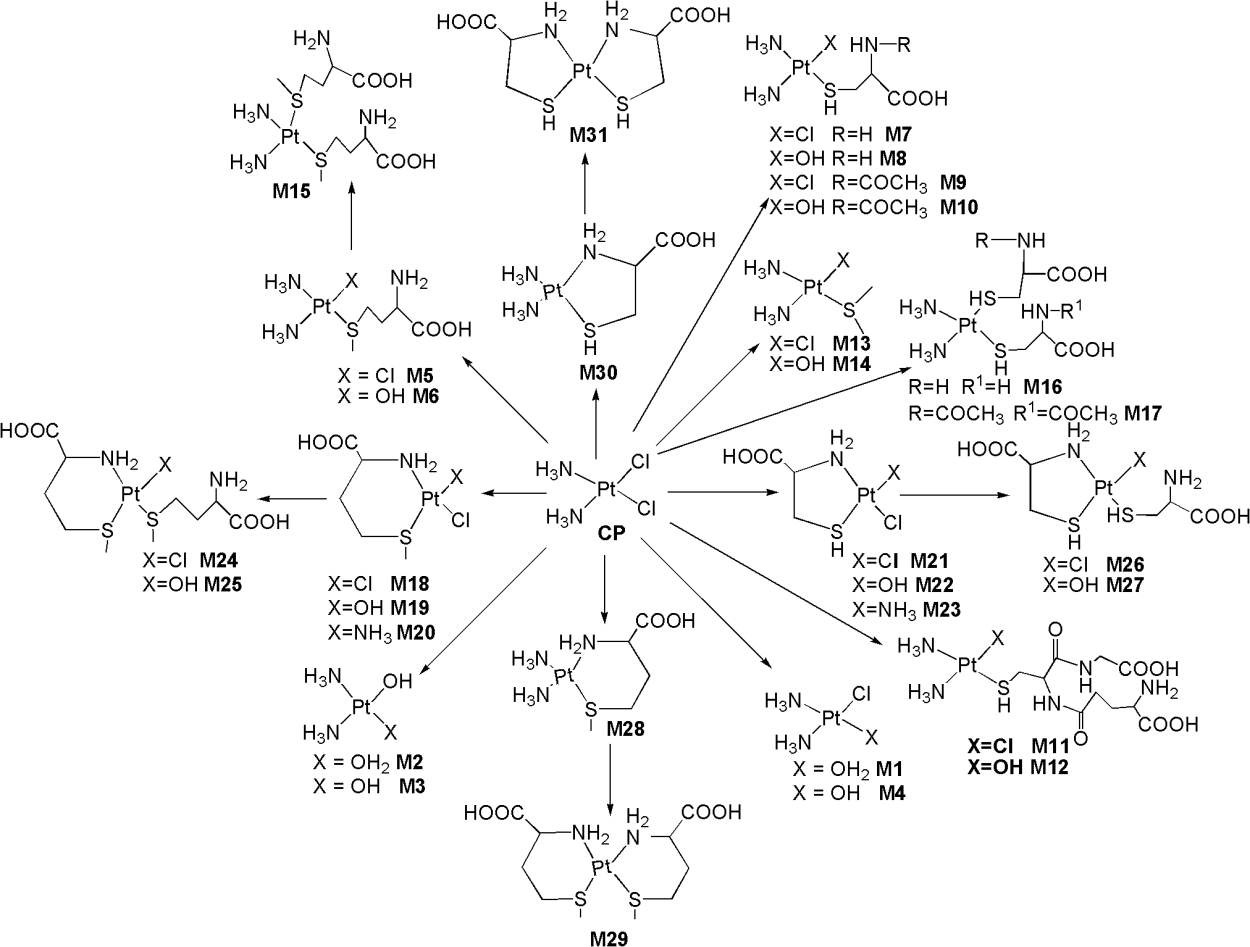
Proposed *in vivo* ligand exchange biotransformation pathway of CP.

Fig 1 shows the LC/ESI-MS-TIC of metabolites of CP formed in kidney tissue homogenate samples. All the metabolites (**M1-M31)** were eluted within 35 min on C-18 column and they were well resolved from parent drug as well as from each other. The positive ion ESI-MS spectrum of **CP** gives moderately abundant [M+H]^+^, [M+NH_4_]^+^ ions and abundant [M+Na]^+^ ion, and low abundance [2M+H]^+^, [2M+NH_4_]^+^ [2M+Na]^+^ ions, whereas the positive ion ESI-MS spectra of all metabolites give abundant [M+H]^+^ ions and moderately abundant [M+Na]^+^ ions. To elucidate the structures of metabolites, we examined the fragmentation of the [M+H]^+^ ions of **CP** and its metabolites by using online LC/ESI-MS/MS experiments in combination with accurate mass measurements. Further, online HDX LC-MS/MS experiments were also carried out to support the structural identification of metabolites. The product ions of all the metabolites were compared with the product ions of **CP**, to assign most probable structures for the observed metabolites (Fig 2). The elemental compositions of thirty one metabolites and their majority of product ions have been confirmed by accurate mass measurements data.

To perform online HDX LC-MS experiments, deuterated solvents (Deuterated water and deuterated methanol) are used as a mobile phase instead of normal mobile phase (deionized water and methanol). The HDX experiments are performed by using D_2_O as sheath liquid for the LC-MS analysis of drug metabolites. They are useful for determining the numbers and positions of exchangeable hydrogens present in the metabolites to characterize them [13]. The exchangeable hydrogen atoms are usually bound to N-, O- and S- atoms in different functional groups such as-NH-,-NH_2_,-OH,-COOH and-SH [11]. The examination of the mass shift of the deuteriated molecule from that of the protonated molecule allow the total number of exchangeable protons to be determined. Interpretation of the product ion spectra helps to determine the location of the exchanged protons and assignment of the site(s) of modification for each metabolite. The structure elucidation of metabolites can be effectively achieved by using deuterated precursor ions compared to protonated precursor ions where the protonated precursor ions do not give accurate information in characterizing the metabolites. Thus, by using HDX, some of the unambiguous metabolite identification, which can be very useful in the early stages of drug discovery, can be achieved without the use of large scale preparation for NMR or other analytical characterizations. We have carried out these HDX experiments to further support the structural characterization of in vivo drug metabolites formed in rat kidney tissues. In this study, the HDX experiments allowed us to distinguish the hydroxylated CP metabolites from other metabolites as well as methionine, cysteine and acetylcysteine straight chain containing CP metabolites from their respective chelated ring containing CP metabolites with the help of numbers and positions of exchangeable hydrogens. Furthermore, these HDX studies also allowed us to identify some metabolites that have the same nominal mass and identical elemental compositions which is not possible to characterize by normal LC-MS/MS spectra.

### Characterization of CP and its metabolites using LC/ESI-MS/MS experiments

#### MS/MS of protonated CP (*m/z* 300.9620)

**Fig 3:**
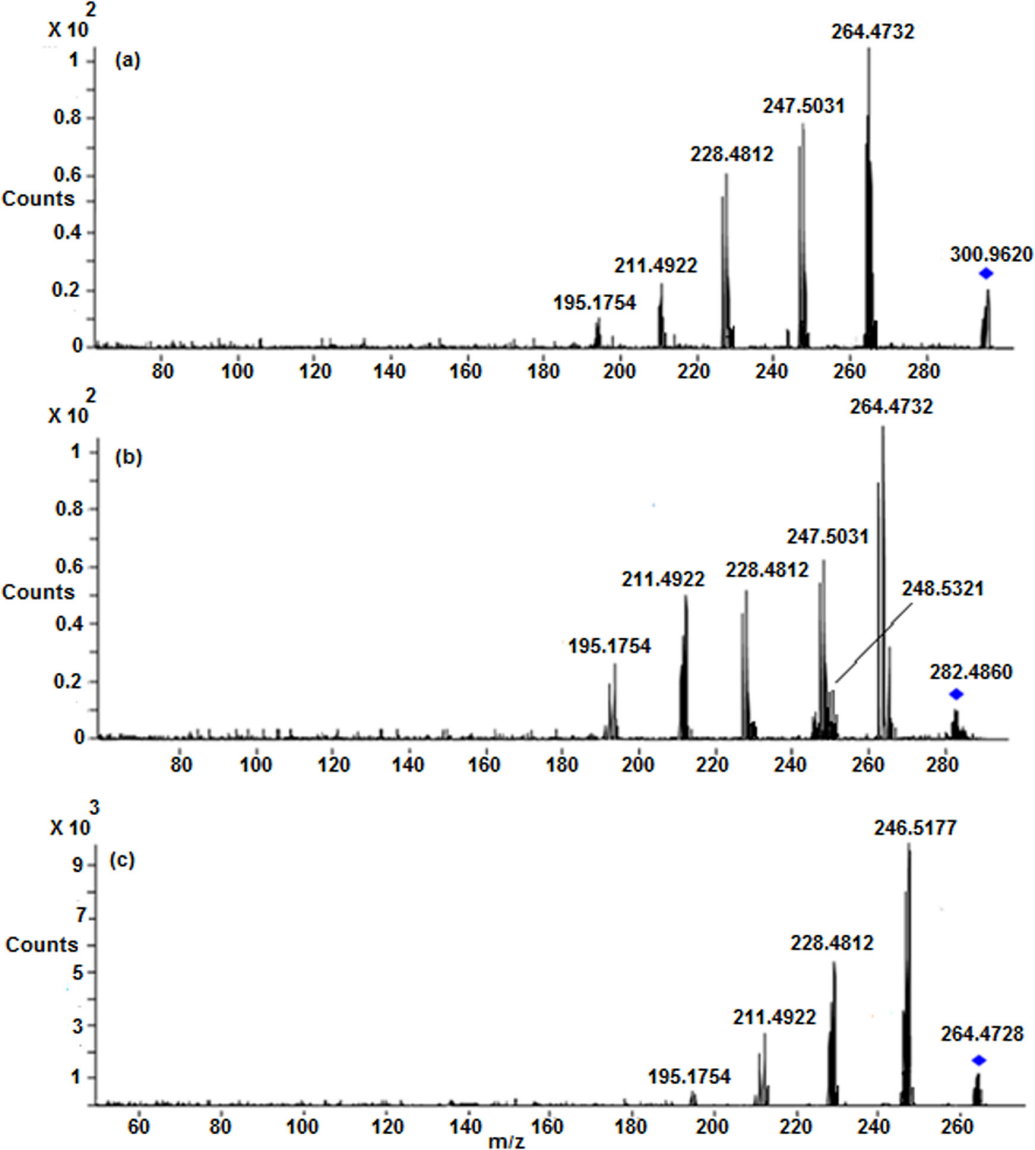
LC/ESI-MS/MS spectra of (a) Protonated CP (b) M1 and (c) M2 at 30 eV.

**Fig 4:**
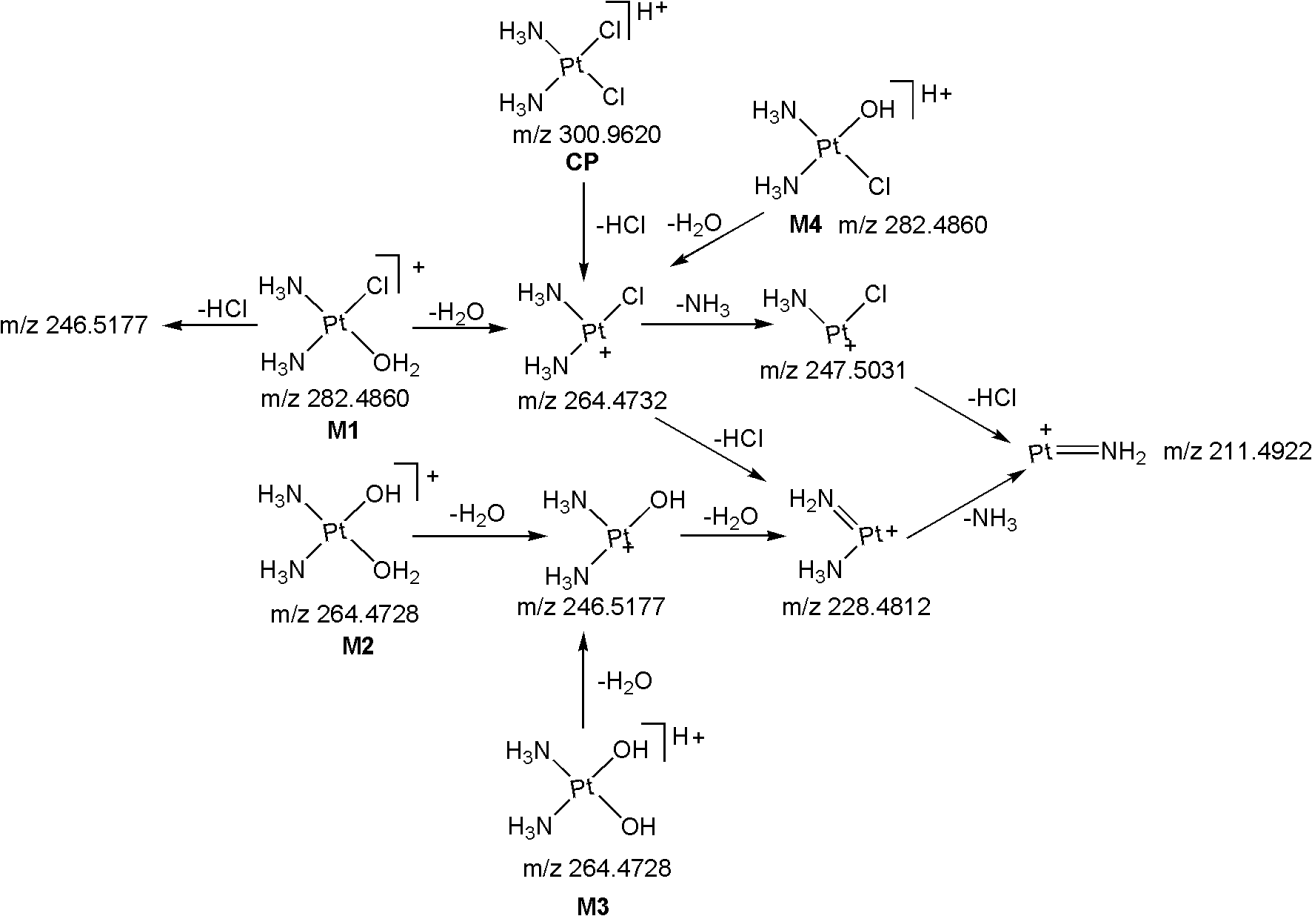
Proposed fragmentation mechanism for CP and its metabolites M1-M4.

The ESI-MS/MS spectrum of [M+H]^+^ ion (*m/z* 300.9620) of CP displays abundant product ions at m/z 264.4732 (loss of HCl), m/z 247.5031 (loss of NH_3_ from m/z 264.4732), m/z 228.4812 (loss of HCl from m/z 264.4732) and low abundance product ions at m/z 211.4922 (loss of HCl from m/z 247.5031) and m/z 195.1754 (platinum cation) (Fig 3A) (Fig 4). It can be noted that the product ions observed in the MS/MS spectrum are consistent with the presence of two chlorine atoms and two ammine groups attached to platinum. All the above fragmentation pathways have been confirmed by MS^n^ experiments and most probable structures are proposed for the product ions based on elemental compositions derived from accurate mass measurements.

#### MS/MS of metabolites of CP

*M1 (m/z 282*.*4860)*: The most abundant metabolite **M1** at *m/z* 282.4860 with an elemental composition of PtN_2_H_8_OCl (Error: 1.92 ppm), was eluted at 4.8 min. The elemental composition data showed that **M1** lacks one chlorine atom and inclusion of one aqua molecule as compared to **CP**, suggesting that it is a mono aqua CP metabolite. To elucidate the structure of metabolite **M1**, the LC/ESI-MS/MS of **M1** was examined (Fig 3B). The CID spectrum of **M1** (Fig 3B) gives the base peak at m/z 264.4732 by the loss of H_2_O as evidenced by its HRMS data (PtN_2_H_6_Cl; 2.41 ppm), clearly confirms the presence of aqua molecule in **M1** (Fig 4). This is also supported by the fact that protonated **CP** loses HCl to form the base peak at m/z 264.4732 (Fig 3A) (Fig 4). In addition, the MS/MS spectrum shows other product ions at m/z 247.5031, m/z 228.4812, m/z 211.4922 and m/z 195.1754 (Fig 3B and Fig 4) which were also observed in the MS/MS of protonated **CP** (Fig 3A) (Fig 4), supports the proposed structure for **M1**. It was reported earlier that **CP** forms major metabolite, mono aqua **CP** [17, 18], based on which, the structure of **M1** can be assigned as a mono aqua **CP** metabolite.

*M2 (m/z 264*.*4728)*: The metabolite **M2** at *m/z* 264.4728 having the elemental composition of PtN_2_H_9_O_2_ (3.21 ppm), was detected at 2.7 min. Its online HDX LC-MS spectrum shows the deuterated molecular ion at *m/z* 265.4944 with an elemental composition of PtN_2_H_8_O_2_D, suggesting the presence of one exchangeable hydrogen (Pt-OH group) in its structure. These elemental compositions data revealed that **M2** lacks two chlorine atoms when compared to **CP**, indicating that it is a des-chlorinated **CP** metabolite. The LC-MS/MS spectrum (Fig 3C) of **M2** shows abundant product ions at *m/z* 246.5177 (PtN_2_H_7_O; 1.88 ppm) and m/z 228.4812 (PtN_2_H_5_; 3.08 ppm) formed by the loss of one and two water molecules (Fig 4), respectively, from **M2**, unlike the LC-MS/MS of protonated **CP** and **M1** [which show the characteristic fragment ions at *m/z* 264.4732 and m/z 247.5031 (Fig 4) diagnostic for the presence of chlorine atom], clearly indicating the presence of hydroxy group (Pt-OH) and aqua molecule (Pt-OH_2_) in the structure of **M2.** Based on these data, the structure of **M2** was characterized to be a mono hydroxylated mono aqua CP metabolite.

*M3 ([M+H]* ^*+*^ *; m/z 264*.*4728)*: The low level metabolite **M3** at *m/z* 264.4728 ([M+H]^+^; PtN_2_H_9_O_2_; 3.21 ppm) got eluted at 1.7 min. Its deuterated molecular ion at *m/z* 267.4932 (PtN_2_O_2_H_6_D_3_) indicates the presence of three exchangeable hydrogens (two hydroxy groups and mobile proton) in its structure. These HRMS data clearly indicate the absence of two chlorine atoms and presence of two hydroxy groups in **M3** as compared to **CP**. Based on these data, **M3** could be a dihydoxylated metabolite of **CP**. This can be seen from the LC-MS/MS spectrum of protonated **M3** which shows abundant product ions at *m/z* 246.5177 and m/z 228.4812 formed by the loss of one and two water molecules (Fig 4), respectively.

*M4 ([M+H]* ^*+*^ *; m/z 282*.*4860)*: The metabolite **M4** at *m/z* 282.4860 ([M+H]^+^; PtN_2_H_8_OCl; 1.92 ppm) was eluted at 3.3 min. Its deuterated molecular ion at *m/z* 284.5132 with an elemental composition of PtN_2_H_6_OClD_2_ indicates the presence of two exchangeable hydrogens (Pt-OH group and mobile proton) in its structure. The elemental composition data showed that **M4** lacks one chlorine atom and inclusion of one hydroxy group as compared to **CP**, suggesting that it is a mono hydroxylated CP metabolite. The LC-MS/MS spectrum of protonated **M4** gives the base peak at m/z 264.4732 (PtN_2_H_6_Cl; 2.41 ppm) by the loss of H_2_O as evidenced by its HRMS data, confirms the presence of hydroxy group (Pt-OH) in **M4** (Fig 4). This is also supported by the fact that protonated **CP** and **M1** lose HCl and H_2_O, respectively, to form the base peak at m/z 264.4732 (Fig 4). Further support comes from the appearance of other structure indicative product ions at m/z 247.5031, m/z 228.4812, m/z 211.4922 and m/z 195.1754 which were already discussed in protonated **CP** and **M1**.

It is worthy to note that the online HDX experiments used in this study allowed us to distinguish the metabolites, **M1-M4** from each other. The metabolites, **M1 & M4** (m/z 282.4860) and **M2 & M3** (m/z 264.4728) that have the same nominal mass and identical elemental compositions were unambiguously distinguished and characterized by HDX LC-MS/MS experiments. These metabolites were distinguished from each other mainly based on the presence of numbers of exchangeable hydrogens in their structures that were different for each metabolite. In HDX experiments, **M4** showed its deuterated molecular ion at m/z 284.5132 (two exchangeable hydrogens; hydroxy group & mobile proton), whereas **M1** detected at m/z 282.4860 (No exchangeable hydrogens). Similarly, the online HDX LC-MS spectrum of **M3** showed its deuterated molecular ion at m/z 267.4932 with three exchangeable hydrogens (two hydroxy groups & mobile proton), whereas **M2** showed it’s deuterated molecular in at m/z 265.4944 with one exchangeable hydrogen (mobile proton). It is important to note that the structures of these metabolites (**M1-M4)** were proposed mainly based on HDX LC-MS experiments. The comparison of the product ion spectra obtained using the precursor ions formed from ‘with’ and ‘without’ HDX experiments clearly indicate that the structure elucidation of hydroxylated metabolites can be effectively achieved by using deuterated precursor ions where the protonated precursor ions have not been given accurate information to characterize them. Besides, these HDX experiments were also helped to further support the structural elucidation of some other metabolites (identified in this work) that has been discussed in appropriate places in the succeeding text.

**Fig 5:**
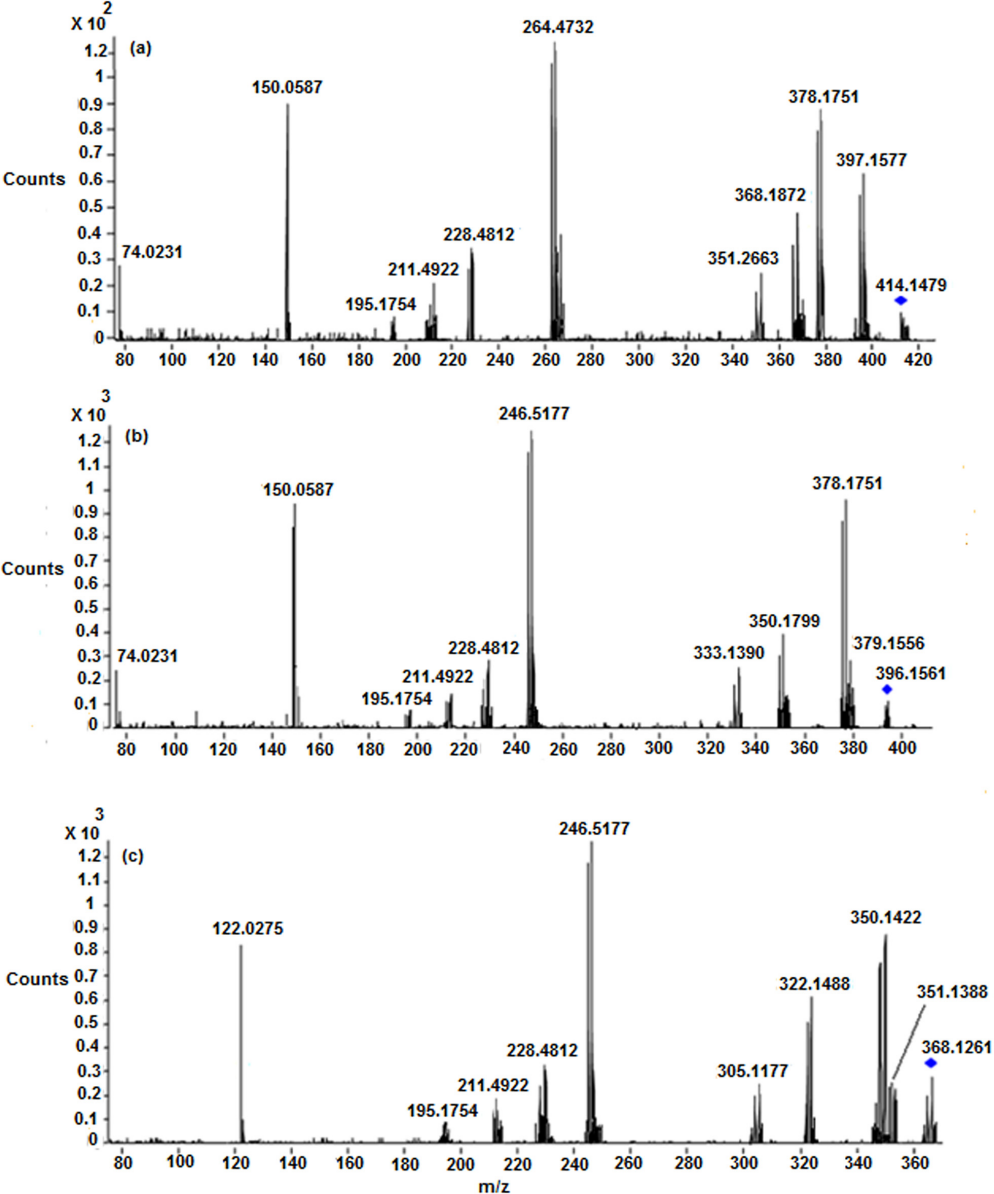
LC/ESI-MS/MS spectra of (a) Protonated M5 (b) Protonated M6 and (c) Protonated M8 at 33 eV.

**Fig 6:**
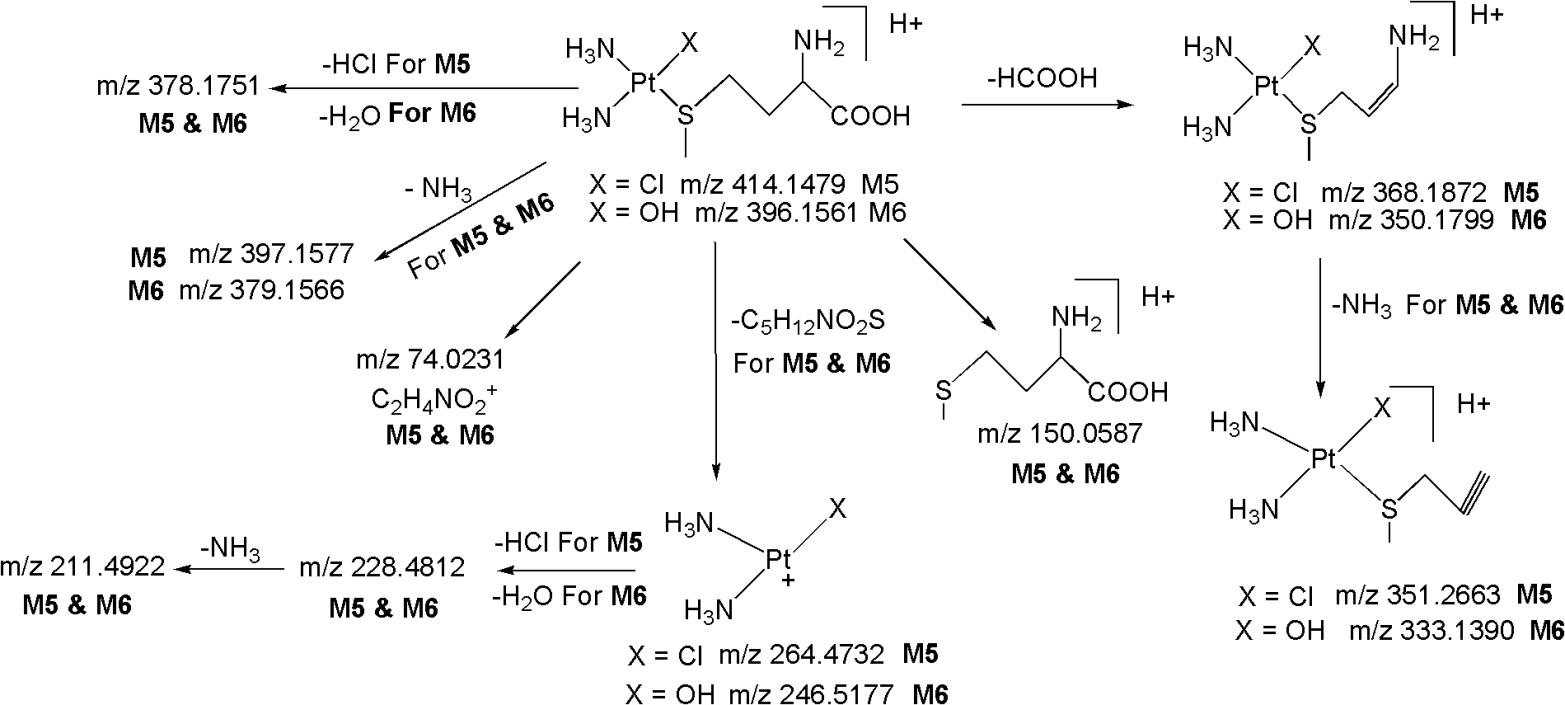
Proposed fragmentation mechanism for metabolites M5 and M6.

*M5 ([M+H]* ^*+*^ *;m/z 414*.*1479)*: The metabolite **M5** at *m/z* 414.1479 ([M+H]^+^; C_5_H_18_ClN_3_O_2_PtS; 3.22 ppm) got eluted at 25.2 min. The appearance of diagnostic product ion at m/z 264.4732 (base peak) (Fig 5A) corresponding to the loss of C_5_H_12_NO_2_S (Fig 6) in the LC-MS/MS spectrum of protonated **M5**, which is also a characteristic product ion observed in the LC-MS/MS of protonated **CP**, **M1** and **M4** (Fig 4), clearly indicating the presence of Pt^+^(NH_3_)_2_(Cl) moiety [two ammine groups, one chlorine atom and platinum atom] in its structure. The LC-MS/MS spectrum of protonated **M5** shows diagnostic product ion at m/z 150.0587 corresponding to protonated methionine, confirms the presence of methionine moiety in its structure. This was further substantiated by MS/MS experiments on deuterated derivative **M5** in which the m/z 150.0587 ion got shifted to m/z 154.0816 due to incorporation of four deuterium atoms (-NH_2_,-COOH and mobile proton). It can be noted that the neutral loss fragments of C_5_H_12_NO_2_S (m/z 264.4732) and HCl (m/z 378.1751) (Fig 6) from the protonated molecular ion, also substantiates the presence of methionine moiety and chlorine atom, respectively in **M5**. Besides, the LC-MS/MS spectrum of protonated **M5** shows other fragment ions at m/z 368.1872 (loss of HCOOH), m/z 351.2663 (loss of NH_3_ from m/z 368.1872) and m/z 74.0231 (C_2_H_4_NO_2_^+^), supports the presence of methionine moiety in its structure. Based on these data, **M5** was identified as a methionine metabolite of **CP**.

*M6 ([M+H]* ^*+*^ *; m/z 396*.*1561)*: The metabolite **M6** at *m/z* 396.1561 ([M+H]^+^; C_5_H_19_N_3_O_3_PtS; -1.88 ppm) was eluted at 19.6 min. The HRMS data revealed that **M6** lacks a chlorine atom and inclusion of hydroxy group as compared to **M5**, suggesting that it is a hydroxylated methionine CP metabolite. The appearance of significant product ion at *m/z* 246.5177 (base peak) (Fig 5B) corresponding to the loss of C_5_H_12_NO_2_S (Fig 6) in the LC-MS/MS spectrum of protonated **M6**, which is also a characteristic ion observed in the LC-MS/MS of protonated **M2** (Fig 3B) (Fig 4), indicating the presence of Pt^+^(NH_3_)_2_(OH) moiety (two ammine groups, one hydroxy group and platinum atom) in the structure of **M6**. This was further supported by the MS/MS spectrum of deuterated **M6** in which the m/z 246.5177 ion got shifted to m/z 247.5244 due to incorporation of one deuterium atom (Pt-OH→Pt-OD). Further, protonated **M6** yields the m/z 378.1751 (C_5_H_17_N_3_O_2_PtS; -2.21 ppm) ion by the loss of H_2_O as evidenced by its HRMS data which clearly shows that H_2_O is eliminated from hydroxy group that is directly attached to platinum atom (Pt-OH) and not from the-COOH group of methionine moiety. This is also supported by the fact that protonated **M5** loses HCl to form *m/z* 378.1751 (Fig 6). Similarly to **M5**, the formation of fragment ions at m/z 150.0587 (protonated methionine) (Fig 5B), m/z 154.0816 (deuterated methionine) and the neutral loss of C_5_H_12_NO_2_S (m/z 246.5177) (Fig 6) from the protonated molecular ion, reflects the presence of methionine moiety in the structure of **M6**. In addition, protonated **M6** yields other product ions at m/z 350.1799 (loss of HCOOH), m/z 333.1390 (loss of NH_3_ from m/z 350.1799) and m/z 74.0231 (C_2_H_4_NO_2_^+^), supports the presence of methionine moiety and hydroxy group in **M6**.

**Fig 7:**
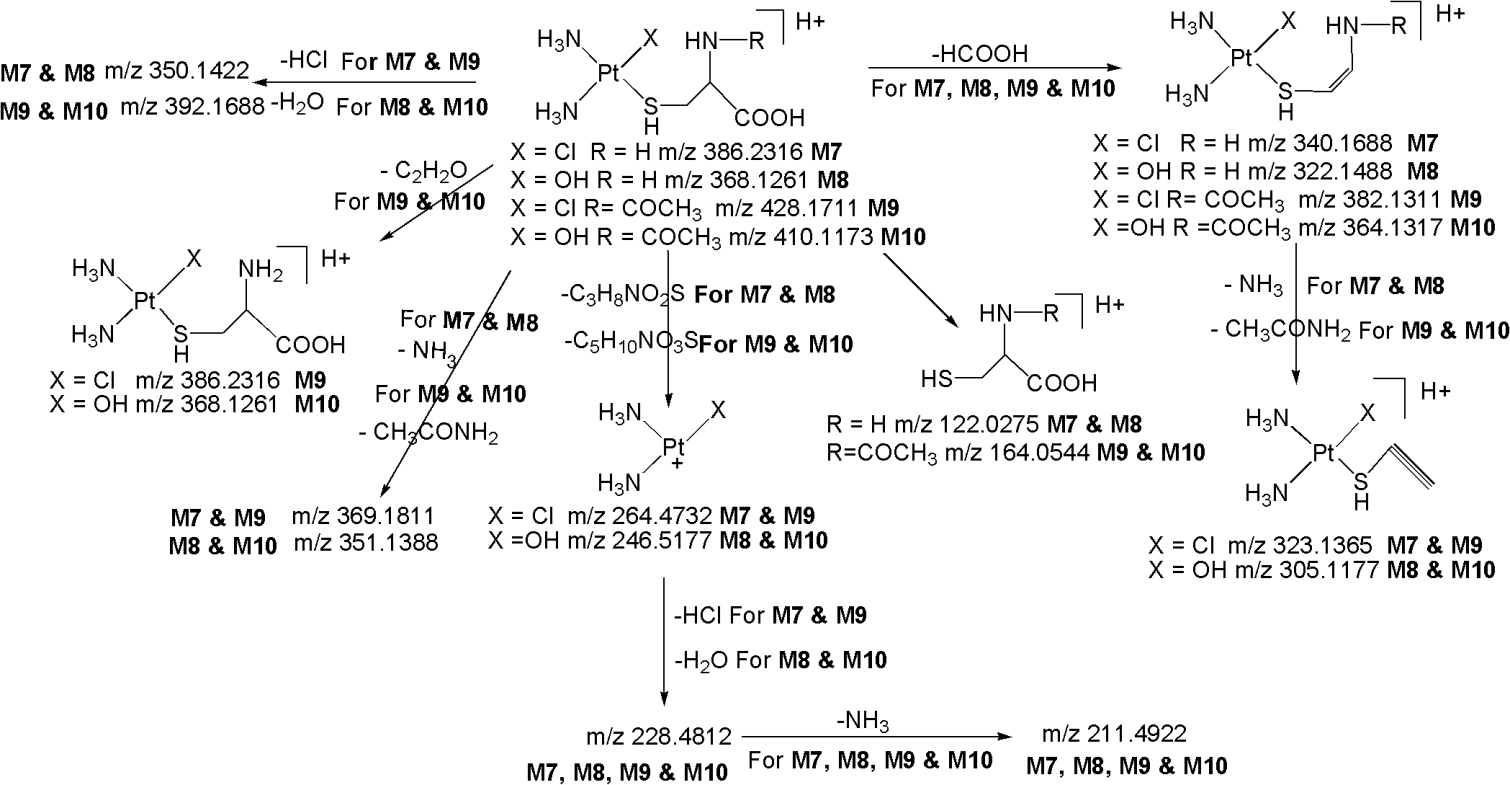
Proposed fragmentation mechanism for metabolites M7-M10.

*M7 ([M+H]* ^*+*^ *; m/z 386*.*2316)*: The metabolite **M7** at *m/z* 386.2316 ([M+H]^+^; C_3_H_14_N_3_O_2_PtSCl; 3.15 ppm) was eluted at 23.5 min. Similarly to protonated **CP, M1, M4** and **M5**, the LC-MS/MS spectrum of protonated **M7** displays the diagnostic product ion at m/z 264.4732 (base peak) corresponding to the loss of C_3_H_8_NO_2_S (Fig 7), authenticating the presence of Pt^+^(NH_3_)_2_(Cl) moiety in its structure. The protonated **M7** yields diagnostic product ion at m/z 122.0275 corresponding to protonated cysteine, confirms the presence of cysteine moiety in its structure. This was further confirmed by the MS/MS of deuterated **M7** in which m/z 122.0275 ion got shifted to m/z 127.0576 due to incorporation of five deuterium atoms (-NH_2_,-COOH,-SH and mobile proton). Further, the neutral loss fragments C_3_H_8_NO_2_S (m/z 264.4732) and HCl (m/z 350.1422) from the protonated molecular ion (Fig 7), substantiate the presence of cysteine moiety and chlorine atom, respectively, in **M7**. Besides, the spectrum also shows other ions at m/z 340.1688 (loss of HCOOH) and m/z 323.1365 (loss of NH_3_ from m/z 340.1688) which are consistent with the proposed structure. Based on these data, **M7** was identified as a cysteine metabolite of CP.

*M8 ([M+H]* ^*+*^ *; m/z 368*.*1261)*: The metabolite **M8** at *m/z* 368.1261 ([M+H]^+^; C_3_H_15_N_3_O_3_PtS; -3.22 ppm) was detected at 18.5 min. The HRMS data revealed that **M8** lacks a chlorine atom and inclusion of hydroxy group as compared to **M7**, suggesting that it is a hydroxylated cysteine metabolite of CP. Similarly to **M2** and **M6**, the LC-MS/MS spectrum of protonated **M8** shows significant product ion at m/z 246.5177 formed by the loss of C_3_H_8_NO_2_S (Fig 5C) (Fig 7), indicating the presence of Pt^+^(NH_3_)_2_(OH) moiety in its structure. This was further supported by the MS/MS spectrum of deuterated **M8** in which the m/z 246.5177 ion got shifted to m/z 247.5244 due to incorporation of one deuterium atom (Pt-OH→Pt-OD). Further, the protonated **M8** yields m/z 350.1422 (C_3_H_13_N_3_O_2_PtS; 2.12 ppm) ion by the loss of H_2_O as evidenced by its HRMS data which clearly shows that H_2_O is eliminated from hydroxy group that is directly attached to platinum atom (Pt-OH) (discussed in **M6**) and not from the-COOH group of cysteine moiety. This is also supported by the fact that protonated **M7** loses HCl to form the base peak at *m/z* 350.1422 (Fig 7). Similarly to **M7**, the formation of diagnostic fragment ions at m/z 122.0275 (protonated cysteine) and m/z 127.0576 (deuterated cysteine), and the neutral loss of C_3_H_8_NO_2_S (m/z 246.5177) (Fig 7) from the protonated molecular ion, reflects the presence of cysteine moiety in the structure of **M8**. Besides, the LC-MS/MS spectrum of protonated **M8** shows other product ions at m/z 322.1488 (loss of HCOOH) and m/z 305.1177 (loss of NH_3_ from m/z 322.1488) which are compatible with the proposed structure.

*M9 ([M+H]* ^*+*^ *; m/z 428*.*1711)*: The metabolite **M9** at *m/z* 428.1711 ([M+H]^+^) with an elemental composition of C_5_H_16_N_3_O_3_PtSCl (3.66 ppm) was eluted at 24.2 min, suggesting an addition of acetyl group to **M7**. This can be seen from the LC-MS/MS spectrum of protonated **M9** which clearly displays an abundant product ion at m/z 386.2316 (protonated **M7**) formed by the loss of C_2_H_2_O (Fig 7). Further, it can be seen from Fig 7 that the neutral loss of CH_3_CONH_2_ (m/z 369.1811) from the protonated molecular ion (*m/z* 428.1711), also confirms the presence of N-acetyl group in **M9**, as this is not the case for protonated **M7** and **M8** which eliminates NH_3_ due to the presence of-NH_2_ group. Similarly to protonated **CP**, **M1, M4, M5** and **M7**, the appearance of significant product ion at m/z 264.4732 which involves the loss of C_5_H_10_NO_3_S (Fig 7), substantiates the presence of Pt^+^(NH_3_)_2_(Cl) moiety in the structure of **M9**. It can be noted that the formation of product ion at m/z 164.0544 (protonated acetylcysteine) and its corresponding deuterated species at m/z 168.0622 (-NH,-SH,-COOH and mobile proton), and the neutral loss of C_5_H_10_NO_3_S (m/z 264.4732) (Fig 7) from the protonated molecular ion, substantiates the presence of acetylcysteine moiety in the structure of **M9**. Other product ions at *m/z* 382.1311, *m/z* 323.1365 and *m/z* 392.1688, are also in line with the proposed structure. Based on these data, **M9** was characterized as an acetylcysteine metabolite of CP.

**Fig 8:**
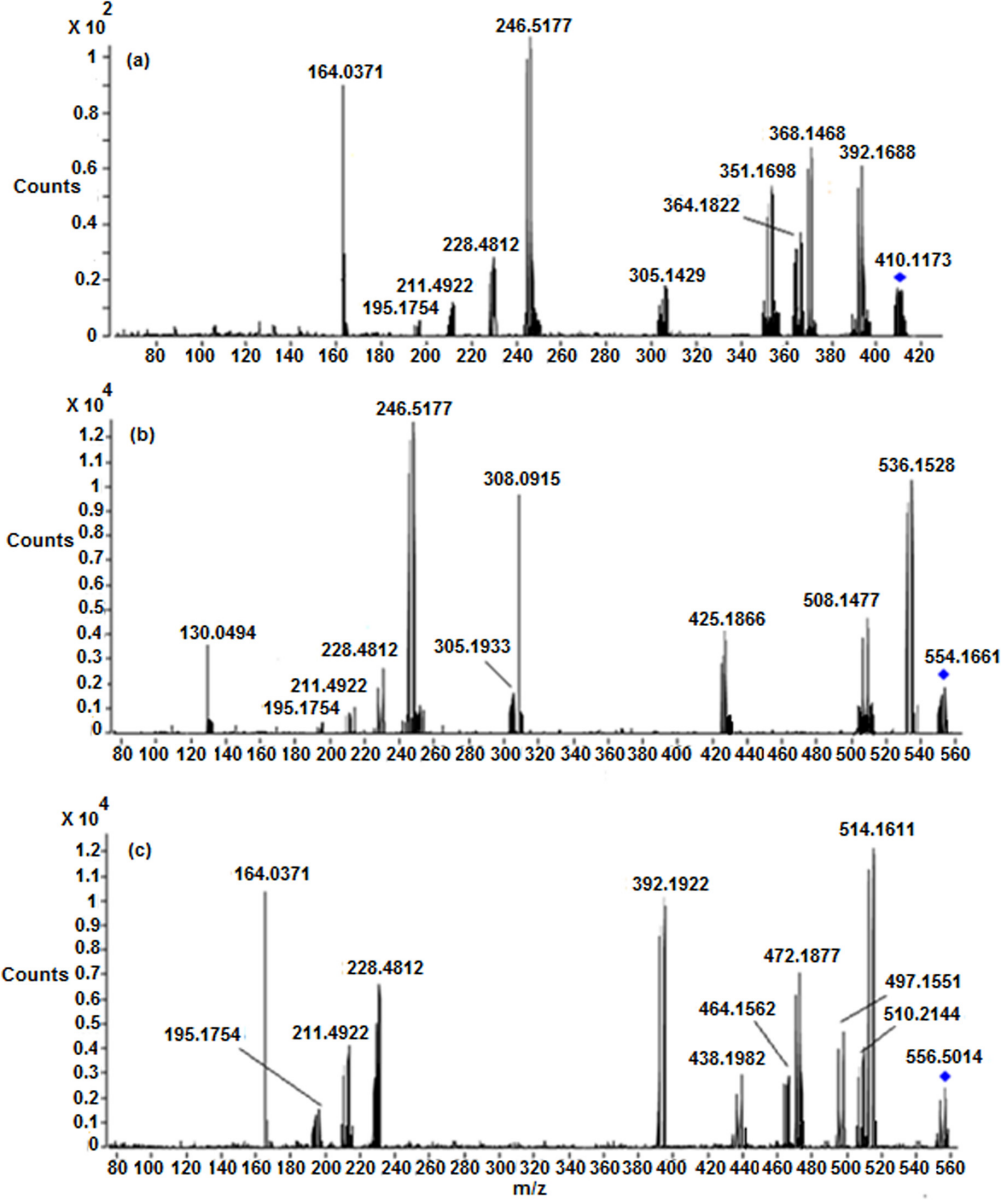
LC/ESI-MS/MS spectra of (a) Protonated M10 (b) Protonated M12 and (c) Protonated M17 at 28 eV.

*M10 ([M+H]* ^*+*^ *; m/z 410*.*1173)*: The metabolite **M10** at *m/z* 410.1173 ([M+H]^+^) with an elemental composition of C_5_H_17_N_3_O_4_PtS (-4.09 ppm) was detected at 22.5 min, suggesting an addition of an acetyl group to **M8**. The HRMS data also revealed the lack of chlorine atom and inclusion of hydroxy group in **M10** when compared to **M9**. The LC-MS/MS spectrum of protonated **M10** gives an abundant peak at m/z 392.1688 (C_5_H_15_N_3_O_3_PtS; -4.09 ppm) (Fig 8A) by the loss of H_2_O, confirms the presence of Pt-OH in **M10**. This is also supported by the fact that protonated **M9** loses HCl to form *m/z* 392.1688. The protonated **M10** also yields m/z 368.1261 ion (Protonated **M8**) which is formed by the loss of C_2_H_2_O (Fig 7), confirms the presence of acetyl group in **M10**. Further, it can be seen from Fig 7 that the loss of CH_3_CONH_2_ (*m/z* 351.1388) from the protonated molecular ion (*m/z* 410.1173) of **M10**, also confirms the presence of N-acetyl group in its structure (Fig 7), as this is not the case for protonated **M7** and **M8** which eliminates NH_3_ due to the presence of-NH_2_ group (Fig 7). The LC-MS/MS spectrum of protonated **M10** also shows other characteristic fragment ions at m/z 246.5177 which involves the loss of C_5_H_10_NO_3_S and m/z 164.0544 (protonated acetylcysteine) (Fig 7), authenticates the presence of Pt^+^(NH_3_)_2_(OH) moiety (discussed in **M2, M6** and **M8)** and acetylcysteine moiety, respectively, in its structure. This was further supported by the MS/MS spectrum of deuterated **M10** in which m/z 246.5177 and m/z 164.0544 ions got shifted to higher masses by one and four units, respectively, due to exchange of labile protons with deuterium atoms.

**Fig 9:**
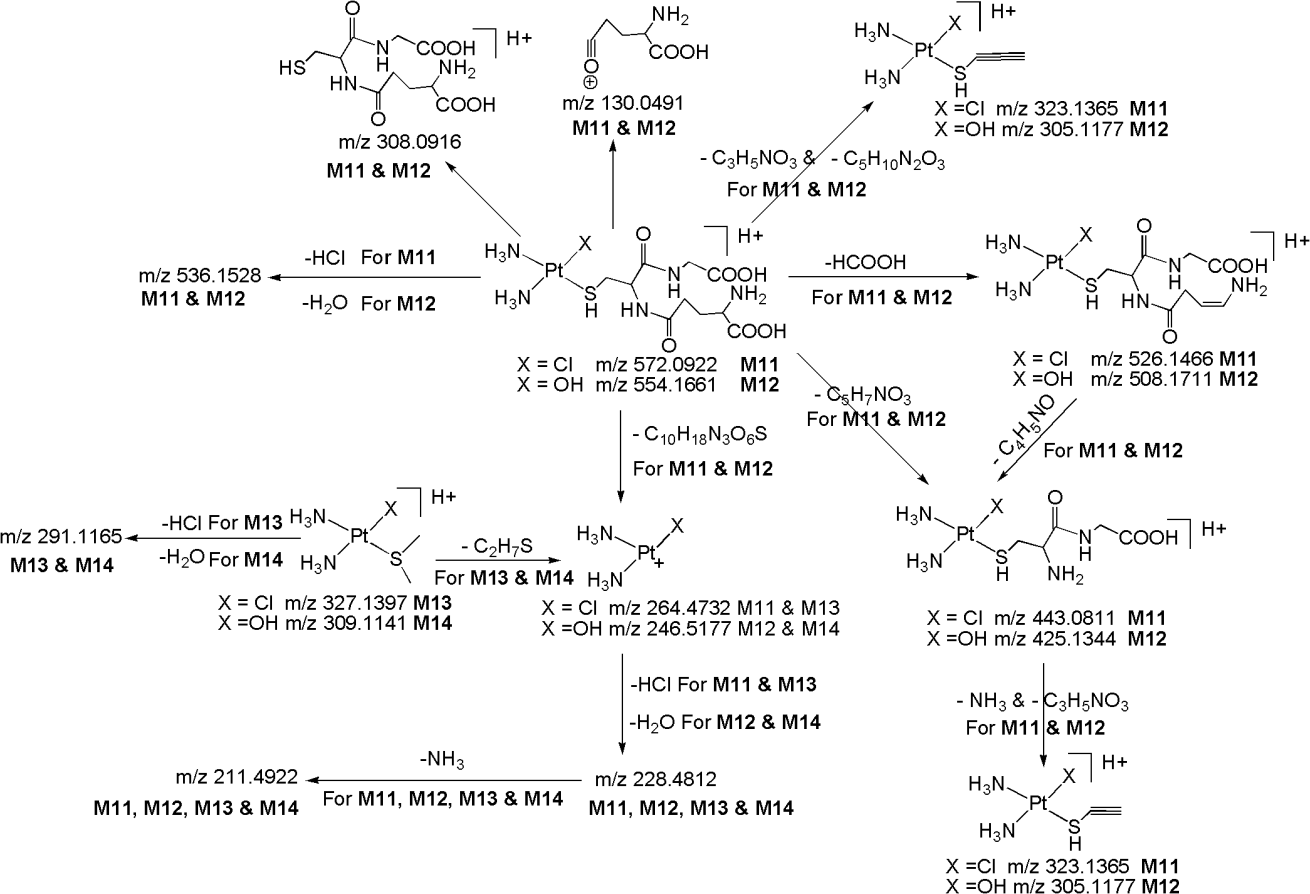
Proposed fragmentation mechanism for metabolites M11-M14.

*M11 ([M+H]* ^*+*^ *; m/z 572*.*0922)*: The metabolite **M11** at *m/z* 572.0922 ([M+H]^+^; C_10_H_24_N_5_O_6_PtSCl; -2.11 ppm) was eluted at 8.2 min. The appearance of diagnostic product ion at m/z 264.4732 due to loss of C_10_H_18_N_3_O_6_S (Fig 9) in the LC-MS/MS spectrum of protonated **M11**, which is also a characteristic product ion observed in the LC-MS/MS of protonated **CP**, **M1, M4, M5, M7** and **M9,** authenticates the presence of Pt^+^(NH_3_)_2_(Cl) moiety in its structure. The protonated **M11** yields diagnostic product ion at m/z 308.0916 corresponding to protonated glutathione, confirms the presence of glutathione moiety in its structure. This was further confirmed by MS/MS of deuterated **M11** in which the m/z 308.0916 ion got shifted to m/z 316.1426 due to incorporation of eight deuterium atoms (-NH_2_, two-NH, two-COOH,-SH and mobile proton). The LC-MS/MS spectrum of protonated **M11** also shows other structure indicative fragment ions at m/z 526.1466 (loss of HCOOH), m/z 443.0811 (loss of C_5_H_7_NO_3_), m/z 323.1365 (simultaneous elimination of C_3_H_5_NO_3_ & C_5_H_10_N_2_O_3_) and m/z 130.0491 (C_5_H_8_NO_3_^+^) which are consistent with the proposed structure for **M11**. Based on these data, **M11** was identified as a glutathione metabolite of **CP**.

*M12 ([M+H]* ^*+*^ *; m/z 554*.*1661)*: The metabolite **M12** at *m/z* 554.1661 ([M+H]^+^; C_10_H_25_N_5_O_7_PtS; 4.41 ppm) was detected at 5.9 min. The HRMS data showed that **M12** lacks a chlorine atom and inclusion of hydroxy group as compared to **M11**, suggesting that it is a hydroxylated glutathione **CP** metabolite. This has been confirmed by the appearance of the Pt^+^(NH_3_)_2_(OH) moiety consisting fragment ion at m/z 246.5177 (discussed in **M2**, **M6, M8** and **M10**) which is formed by the neutral loss of C_10_H_18_N_3_O_6_S and m/z 308.0916 (protonated glutathione) (Fig 9), in the LC-MS/MS spectrum of protonated **M12** (Fig 8B). This was also further confirmed by the MS/MS spectrum of deuterated **M12** in which the above fragment ions got shifted to higher masses due to exchange of labile protons with deuterium *i*.*e*. *m/z* 246.5177→247.5244 and *m/z* 308.0916→316.1426. The protonated **M12** also yields m/z 536.1528 ion (C_10_H_23_N_5_O_6_PtS; 2.51 ppm) by the loss of H_2_O, reflects the presence of hydroxy group (Pt-OH) in its structure which was further supported by the fact that protonated **M11** loses HCl to form *m/z* 536.1528. In line with this, the MS/MS of deuterated **M12** also showed the loss of D_2_O. Further support comes from other structure indicative product ions at m/z 508.1711 (loss of HCOOH), m/z 425.1344 (loss of C_5_H_7_NO_3_), m/z 305.1177 (simultaneous elimination of C_3_H_5_NO_3_ & C_5_H_10_N_2_O_3_) and m/z 130.0491 (C_5_H_8_NO_3_^+^) observed in the LC-MS/MS of protonated **M12**.

*M13 ([M+H]* ^*+*^ *; m/z 327*.*1397)*: The metabolite **M13** at *m/z* 327.1397 ([M+H]^+^) with an elemental composition of C_2_H_13_N_2_PtSCl (-2.88 ppm) was detected at 29.4 min. Similarly to protonated **CP**, **M1**, **M4, M5, M7, M9** and **M11,** the LC-MS/MS spectrum of protonated **M13** shows the characteristic fragment ion at m/z 264.4732 involves the loss of C_2_H_7_S, confirms the presence of Pt^+^(NH_3_)_2_(Cl) moiety (Fig 9) in its structure. From Fig 9, it is notable that the neutral loss fragments C_2_H_7_S (m/z 264.4732) and HCl (m/z 291.1165) from the protonated molecular ion, designate the presence of thioether moiety and chlorine atom, respectively, in **M13**. Based on these data, **M13** was identified as a thioether metabolite of CP.

*M14 ([M+H]* ^*+*^ *; m/z 309*.*1141)*: The metabolite **M14** at *m/z* 309.1141 ([M+H]^+^; C_2_H_14_N_2_OPtS; 1.92 ppm) was detected at 29.1 min. Its deuterated molecular at m/z 311.1171 (C_2_H_12_N_2_OPtSD_2_), suggests the presence of two exchangeable hydrogens in its structure (Pt-OH group and mobile proton). These HRMS data clearly showed that **M14** lacks a chlorine atom and inclusion of hydroxy group as compared to **M13**, suggesting that it is a hydroxylated thioether CP metabolite. This has been confirmed by the appearance of Pt^+^(NH_3_)_2_(OH) moiety consisting fragment ion at m/z 246.5177 (discussed in **M2**, **M6, M8, M10** and **M12**) formed by the loss of C_2_H_7_S and m/z 291.1165 ion which involves the loss of H_2_O from Pt-OH group (discussed in **M6, M8** and **M10**) (Fig 9), in the LC-MS/MS spectrum of protonated **M14**. Further, this has been substantiated by the MS/MS spectrum of deuterated **M14** in which the m/z 246.5177 ion got shifted to m/z 247.5244 ion due to exchange of one labile proton with deuterium atom (Pt-OH)→(Pt-OD) and the deuterated molecular ion (m/z 311.1171) yields the m/z 291.1165 ion by the loss of D_2_O (from Pt-OD and mobile proton).

**Fig 10:**
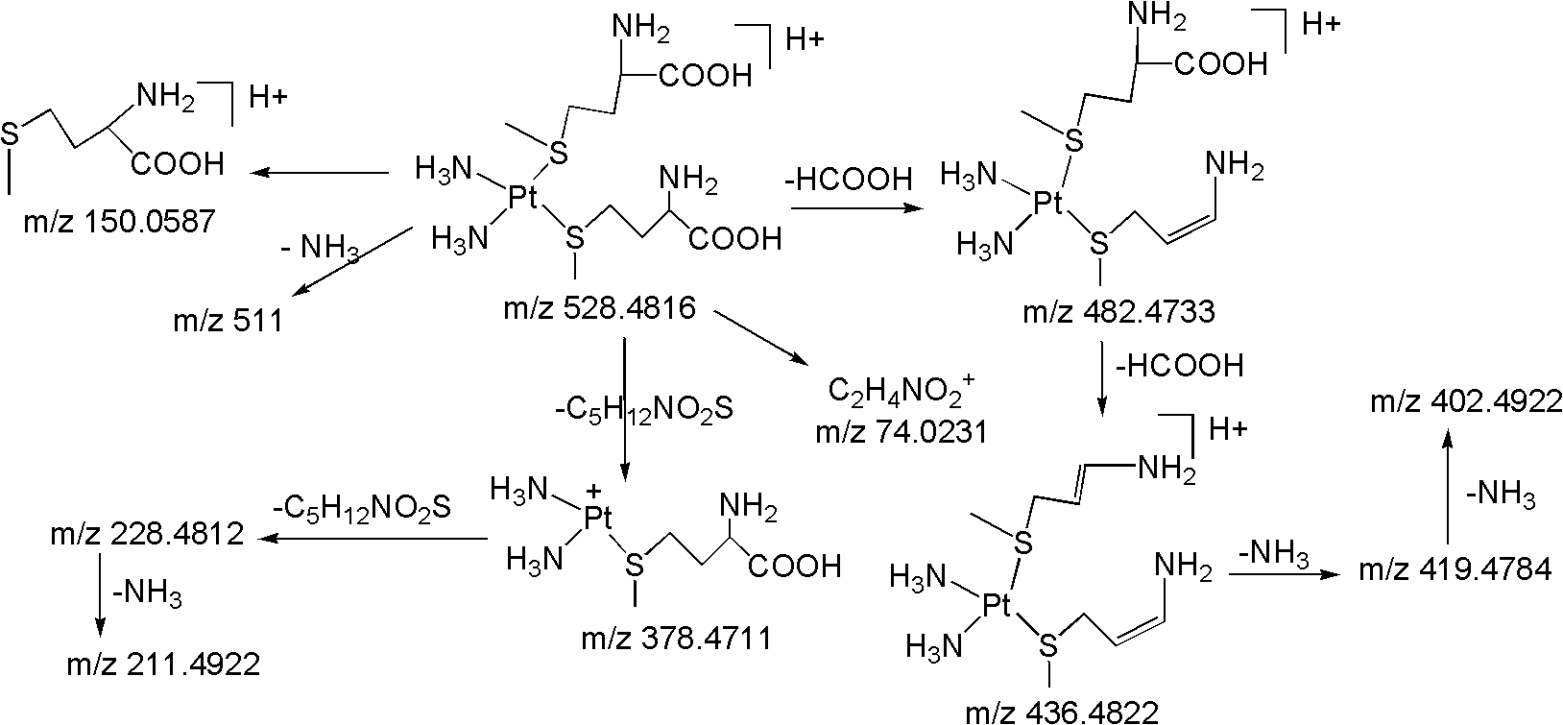
Proposed fragmentation mechanism for metabolite M15.

*M15 ([M+H]* ^*+*^ *; m/z 528*.*4816)*: The second most abundant metabolite **M15** at *m/z* 528.4816 ([M+H]^+^) with an elemental composition of C_10_H_29_N_4_O_4_PtS_2_ (3.96 ppm) was eluted at 14.6 min. The HRMS data suggests the lack of one chlorine atom and presence of an additional methionine moiety in **M15** as compared to **M5**. This can be seen from the LC-MS/MS spectrum of protonated **M15** which shows abundant product ions at m/z 378.4711 and m/z 228.4812 corresponding to a probable loss of one and two methionine moieties, respectively, and m/z 150.0587 (protonated methionine) (Fig 10). It can be noted that the peaks at m/z 264.4732 (Pt^+^(NH_3_)_2_Cl) and m/z 246.5177 (Pt^+^(NH_3_)_2_OH) would have been present and the sequential losses of two neutral species of methionine would have been absent in case of one methionine moiety in protonated **M15** as discussed in **M5** and **M6.** Further support comes from the appearance of moderately abundant product ions at m/z 482.4733 and m/z 436.4822 which were formed by the neutral loss of one and two formic acids, respectively, from protonated **M15.** Similarly to **M5** and **M6**, the appearance of m/z 150.0587 ion (protonated methionine) in the MS/MS of protonated **M15** and m/z 154.0816 ion (deuterated methionine) in the MS/MS of deuterated **M15**, reflects the presence of methionine moiety in the structure of **M15**. Based on these data, the structure of **M15** can be assigned as a di-methionine metabolite of CP.

**Fig 11:**
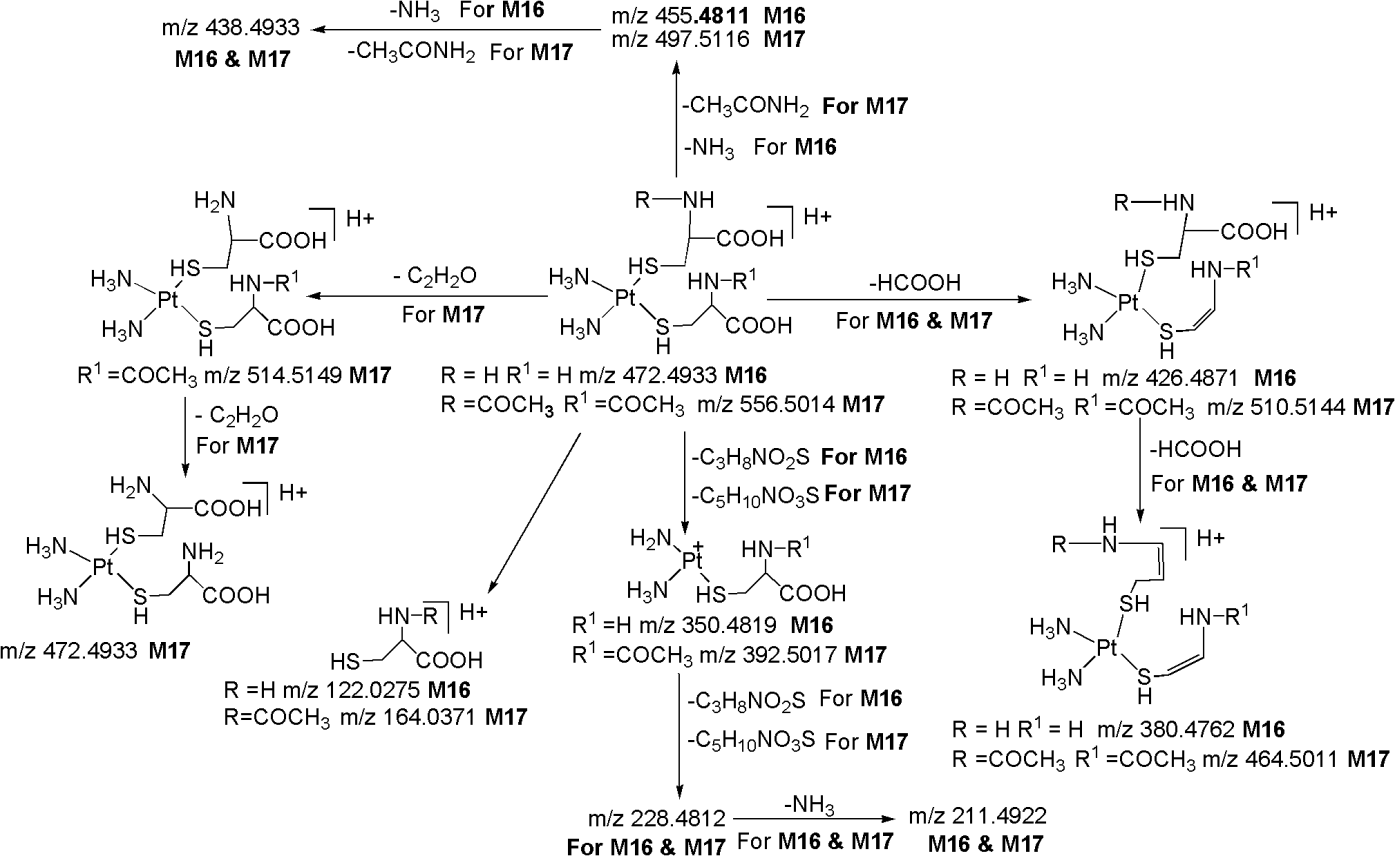
Proposed fragmentation mechanism for metabolites M16 and M17.

*M16 ([M+H]* ^*+*^ *; m/z 472*.*4933)*: The third most abundant metabolite **M16** at *m/z* 472.4933 ([M+H]^+^) with an elemental composition of C_6_H_21_N_4_O_4_PtS_2_ (-2.96 ppm) was eluted at 12.9 min. The HRMS data suggests the lack of one chlorine atom and inclusion of an additional cysteine moiety in **M16** as compared to **M7**. This can be seen from the LC-MS/MS spectrum of protonated **M16** which shows abundant product ions at m/z 350.4819 and m/z 228.4812 corresponding to a probable loss of one and two cysteine moieties, respectively, and m/z 122.0275 (protonated cysteine). The peaks at m/z 264.4732 (Pt^+^(NH_3_)_2_Cl) and m/z 246.5177 (Pt^+^(NH_3_)_2_OH) would have been present and the sequential losses of two neutral species of cysteine would have been absent in case of one cysteine moiety in **M16** as discussed in **M7** and **M8.** The moderately abundant product ions at m/z 426.4871 (loss of HCOOH) and m/z 380.4762 (loss of two HCOOH), also designate the presence of two cysteine moieties in **M16** (Fig 11). Similarly to **M7** and **M8,** the appearance of m/z 122.0275 (protonated cysteine) and m/z 127.0576 (deuterated cysteine) in the MS/MS of protonated and deuterated **M16**, respectively, substantiates the presence of cysteine moiety in the structure of **M16**. Based on these data, **M16** was characterized to be a di-cysteine metabolite of CP.

*M17 ([M+H]* ^*+*^ *; m/z 556*.*5014)*: The protonated metabolite **M17** at *m/z* 556.5014 with an elemental composition of C_10_H_25_N_4_O_6_PtS_2_ (-3.88 ppm) was eluted at 15.7 min. The HRMS data suggests the lack of one chlorine atom and the inclusion of an additional acetylcysteine moiety in **M17** as compared to **M9**. This can be seen from the LC-MS/MS spectrum of protonated **M17** (Fig 8C) which shows abundant product ions at m/z 392.5017 and m/z 228.4812 corresponding to a probable loss of one and two acetylcysteine moieties, respectively and m/z 164.0544 (protonated acetylcysteine). Further support comes from the appearance of significant product ions at m/z 514.5149 and m/z 472.4933 & m/z 497.5116 and m/z 438.4933 & m/z 510.5144 and m/z 464.5011 (Fig 8C) which were formed by the neutral loss fragments of one and two C_2_H_2_O & CH_3_CONH_2_ & HCOOH, respectively, from protonated **M17** (Fig 11). Similarly to **M15** and **M16**, the peaks at m/z 264.4732 ((Pt^+^(NH_3_)_2_Cl)) and m/z 246.5177 ((Pt^+^(NH_3_)_2_OH)) would have been present and the sequential losses of two neutral species of acetylcysteine would have been absent in case of one acetylcysteine moiety in **M17** as discussed in **M9** and **M10.** Similarly to **M9** and **M10**, the appearance of m/z 164.0544 (protonated acetylcysteine) in the MS/MS of protonated **M17** (Fig 8C) and m/z 168.0622 (deuterated acetylcysteine) in the MS/MS of deuterated **M17**, confirms the presence of acetylcysteine moiety in the structure of **M17**. Based on these data, **M17** was identified as a di-acetylcysteine metabolite of CP.

*M18 (m/z 414*.*0922)*: The metabolite **M18** at *m/z* 414.0922 with an elemental composition of C_5_H_11_NO_2_PtSCl_2_ (4.09 ppm) was eluted at 21.6 min. The HRMS data suggests the presence of two chlorine atoms and methionine moiety in the structure of **M18**. The LC-MS/MS spectrum of **M18** shows abundant fragment ions at m/z 378.1412 and m/z 342.1655 due to loss of one and two HCl molecules, respectively, from the molecular ion, confirms the presence of two intact chlorine atoms in its structure. The absence of peak corresponds to loss of HCOOH, which is a major fragmentation observed in the LC-MS/MS of methionine containing metabolites (**M5, M6** and **M15),** clearly eliminates the possibility of straight chain methionine moiety in the structure of **M18.** On the contrary, the appearance of diagnostic fragment ion at m/z 370.1542 corresponding to the loss of CO_2_ from the molecular ion, confirms the presence of chelated methionine ring in **M18**. Further, it can be noted that the ion at m/z 228.4812 (discussed in protonated **CP** and **M1-M17**) would have been present in case of Pt^+^N_2_H_5_ moiety (if the chelated methionine ring does not present in **M18).** Based on these data, **M18** was characterized to be a chelated methionine **CP** metabolite.

*M19 (m/z 396*.*1387)*: The metabolite **M19** at *m/z* 396.1387 with an elemental composition of C_5_H_12_NO_3_PtSCl (3.66 ppm) was eluted at 15.7 min. The HRMS data suggests the lack of one chlorine atom and presence of hydroxy group in **M19** as compared to **M18.** This has been confirmed from the LC-MS/MS spectrum of **M19** which shows abundant fragment ion at m/z 378.1412 (C_5_H_10_ClNO_2_PtS; 1.56 ppm) involves the loss of H_2_O. The m/z 378.1412 ion loses HCl to form m/z 342.1655 ion which confirms the presence of intact chlorine atom in **M19** as discussed in **M18**. Similarly to **M18**, the absence of peak corresponds to loss of HCOOH and appearance of diagnostic fragment ion at m/z 352.1173 formed by the loss of CO_2_ from molecular ion, clearly eliminates the possibility of straight chain methionine moiety and confirms the presence of chelated methionine ring in the structure of **M19**. Further, the absence of m/z 228.4812 (Pt^+^N_2_H_5_) (discussed in protonated **CP** and **M1-M17**), substantiates the presence of the chelated methionine ring in **M19** as similar to **M18.** Based on these data, **M19** was characterized to be a hydroxylated chelated methionine **CP** metabolite.

*M20 (m/z 396*.*4976)*: The metabolite **M20** at *m/z* 396.4976 with an elemental composition of C_5_H_14_N_2_O_2_PtSCl (-3.55 ppm) was eluted at 27.8 min. The elemental composition data indicates the lack of one chlorine atom and presence of ammine group in **M20** as compared to **M18**. This can be seen from the LC-MS/MS spectrum of **M20** which shows significant ion at m/z 379.1012 formed by the loss of NH_3_, has been evidenced by HRMS data (C_5_H_11_ClNO_2_PtS; -4.05 ppm). Further, the m/z 379.1012 ion loses HCl to form m/z 343.1181 ion which confirms the presence of intact chlorine atom in the structure of **M20** as discussed in **M18** and **M19**. Similarly to **M18** and **M19**, the absence of peak corresponds to loss of HCOOH and appearance of diagnostic fragment ion at m/z 352.1157 corresponding to loss of CO_2_ from **M20**, clearly eliminates the possibility of straight chain methionine moiety and confirms the presence of chelated methionine ring in **M20**. Similarly to **M18** and **M19**, the absence of m/z 228.4812 ion (Pt^+^N_2_H_5_) (discussed in protonated **CP** and **M1-M14**), supports presence of the chelated methionine ring in **M20**.

*M21 (m/z 386*.*1451)*: The metabolite **M21** at *m/z* 386.1451 with an elemental composition of C_3_H_7_NO_2_PtS Cl_2_ (2.88 ppm) was eluted at 22.3 min. The HRMS data suggests the presence of two chlorine atoms and cysteine moiety in the structure of **M21**. The LC-MS/MS spectrum of **M21** shows abundant fragment ions at m/z 350.1556 and m/z 314.1277 due to loss of one and two HCl molecules, respectively, from the molecular ion, confirms the presence of two intact chlorine atoms in **M21**. The absence of peak corresponds to loss of HCOOH and the appearance of diagnostic fragment ion at m/z 342.1175 formed by the loss of CO_2_ from molecular ion, clearly eliminates the possibility of straight chain cysteine moiety and substantiates the presence of chelated cysteine ring in **M21**. The absence of m/z 228.4812 ion (Pt^+^N_2_H_5_) (discussed in protonated **CP** and **M1-M17**)**,** also supports the presence of chelated cysteine ring in **M21**. Based on these data, **M21** was characterized to be a chelated cysteine CP metabolite.

*M22 (m/z 368)*: The metabolite **M22** at *m/z* 368.1911 with an elemental composition of C_3_H_8_NO_3_PtSCl (4.03 ppm) was eluted at 17.8 min. The HRMS data suggests the lack of one chlorine atom and presence of hydroxy group in **M22** as compared to **M21**. This can be seen from the LC-MS/MS spectrum of **M22** which shows abundant fragment ion at m/z 350.1556 (C_3_H_6_ClNO_2_PtS; 2.93 ppm) formed by the loss of H_2_O. Further, the m/z 350.1556 ion loses HCl to form m/z 314.1277 ion which confirms the presence of intact chlorine atom in the structure of **M22** as discussed in **M21**. Similarly to **M21**, the absence of peak corresponds to loss of HCOOH and the appearance of diagnostic fragment ion at m/z 324.1866 corresponding to the loss of CO_2_ from **M22,** clearly eliminates the possibility of straight chain cysteine moiety and confirms the presence of chelated methionine ring in **M22**. Similarly to **M18-M21**, the absence of m/z 228.4812 ion (Pt^+^N_2_H_5_) (discussed in protonated **CP** and **M1-M17**)**,** supports the presence of chelated cysteine ring in **M21**. Based on these data, **M22** was characterized to be a hydroxylated chelated cysteine CP metabolite.

*M23 (m/z 368*.*5646)*: The metabolite **M23** at *m/z* 368.5646 with an elemental composition of C_3_H_10_N_2_O_2_PtSCl (-4.77 ppm) was eluted at 28.7 min. The accurate mass measurements data indicates the lack of one chlorine atom and presence of ammine group in **M23** as compared to **M21**. This can be seen from the LC-MS/MS spectrum of **M23** which shows diagnostic fragment ion at m/z 351.1566 formed by the loss of NH_3_ (C_3_H_7_ClNO_2_PtS; -3.32 ppm). Further, the m/z 351.1566 ion loses HCl to form m/z 315.4811 ion which confirms the presence of intact chlorine atom in the structure of **M23** as discussed in **M21** and **M22**. Similarly to **M21** and **M22**, the absence of the peak corresponds to the loss of HCOOH and the appearance of m/z 324.4622 ion due to the loss of CO_2_ from molecular ion, clearly eliminates the possibility of straight chain cysteine moiety and confirms the presence of chelated cysteine ring in **M23**.

**Fig 12:**
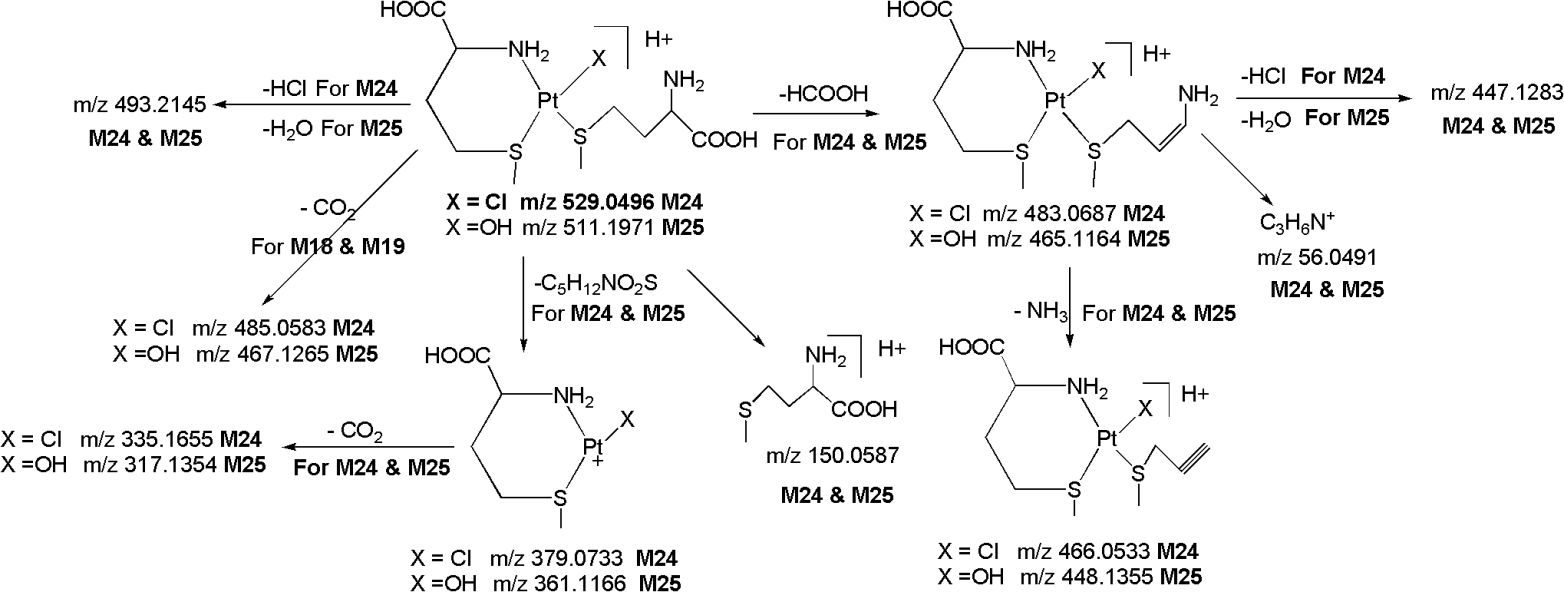
Proposed fragmentation mechanism for metabolites M24 and M25.

*M24 ([M+H]* ^*+*^ *; m/z 529*.*0496)*: The metabolite **M24** at *m/z* 529.0496 ([M+H]^+^) with an elemental composition of C_10_H_23_N_2_O_4_PtS_2_Cl (4.55 ppm) was eluted at 9.1 min. The LC-MS/MS spectrum of protonated **M24** displays abundant product ions at m/z 379.0733 (loss of C_5_H_12_NO_2_S), m/z 483.0687 (loss of HCOOH) and m/z 150.0587 (protonated methionine) (Fig 12), indicates the presence of straight chain methionine in its structure. The formation significant ion at m/z 485.0583 which involves the loss of CO_2_ and absence of the m/z 228.4812 ion (Pt^+^N_2_H_5_) (discussed in protonated **CP** and **M1-M17**), substantiate the presence of one chelated methionine ring in **M24.** Another moderately abundant ion at m/z 493.2145 formed by the loss of HCl (Fig 12), reflects the presence of intact chlorine atom in **M24**.

*M25 ([M+H]* ^*+*^ *; m/z 511*.*1971)*: The metabolite **M25** at *m/z* 511.1971 ([M+H]^+^) with an elemental composition of C_10_H_24_N_2_O_5_PtS_2_ (4.38 ppm) was eluted at 6.6 min. The elemental composition data indicates the lack of one chlorine atom and inclusion of hydroxy group in **M25** as compared to **M24.** This can be seen from the LC-MS/MS spectrum of protonated **M25** which displays an abundant product ion at m/z 493.2145 (C_10_H_22_N_2_O_4_PtS_2_; 3.36 ppm) formed by the loss of H_2_O. This was further supported by the fact that protonated **M24** loses HCl to form the base peak m/z 493.2145. This clearly indicates that H_2_O is eliminated from Pt-OH group and not from the-COOH group of methionine group. In line with this the MS/MS of deuterated **M25** also showed the loss of D_2_O. The protonated **M25** yields abundant product ions at m/z 361.1166 (loss of C_5_H_12_NO_2_S), m/z 465.1164 (loss of HCOOH) and m/z 150.0587 (protonated methionine), indicates the presence of straight chain methionine in its structure. Further, the formation an abundant product ion at m/z 467.1265 (Fig 12) which involves the loss of CO_2_ and absence of the m/z 228.4812 ion (Pt^+^N_2_H_5_) (discussed in protonated **CP** and **M1-M17**), supports the presence of one chelated methionine ring in **M25**.

**Fig 13:**
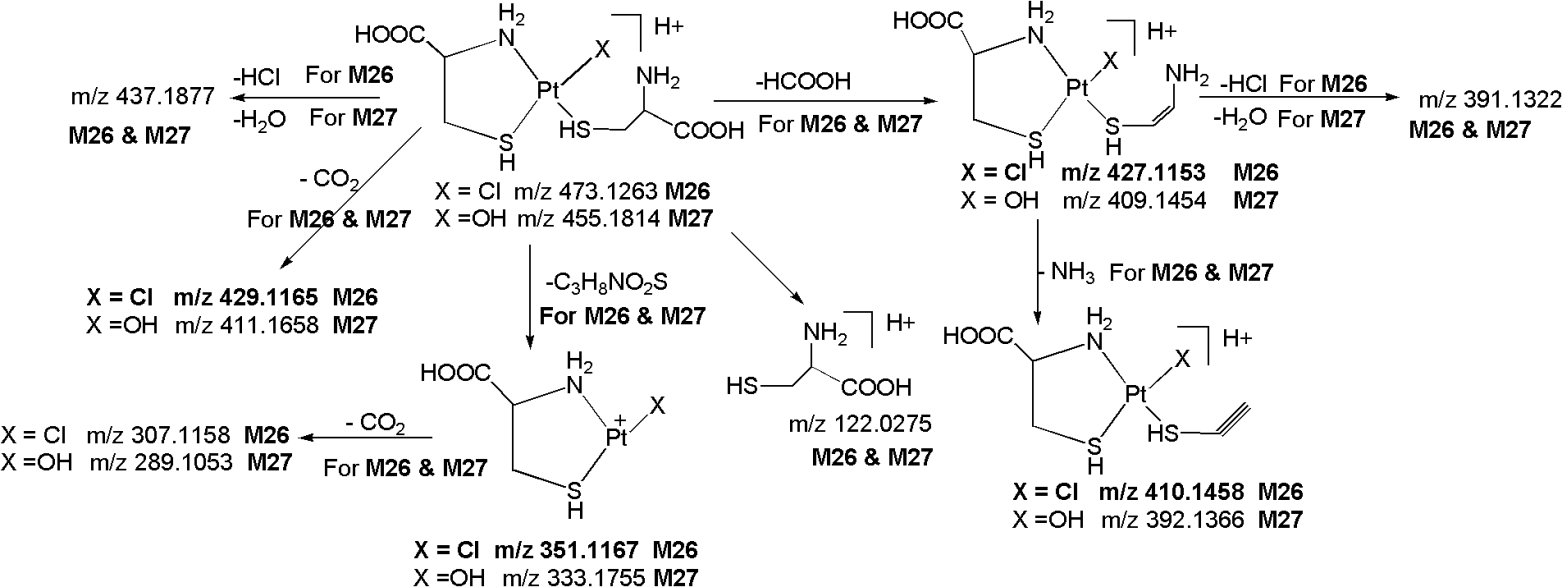
Proposed fragmentation mechanism for metabolites M26 and M27.

*M26 ([M+H]* ^*+*^ *; m/z 473*.*1263)*: The metabolite **M26** at *m/z* 473.1263 ([M+H]^+^; C_6_H_15_N_2_O_4_PtS_2_Cl; 4.11 ppm) was detected at 10.2 min. The LC-MS/MS spectrum of protonated **M26** displays abundant product ions at m/z 351.1167 (loss of C_3_H_8_NO_2_S), m/z 427.1153 (loss of HCOOH) and m/z 122.0275 (protonated cysteine) (Fig 13), demonstrating the presence of strain chain cysteine in **M26**. Further, the formation an abundant ion at m/z 429.1165 which involves the loss of CO_2_ and the absence of m/z 228.4812 ion (Pt^+^N_2_H_5_) (discussed in protonated **CP** and **M1-M17**), confirms the presence of chelated cysteine ring in **M26.** It is notable that the m/z 437.1877 ion which is formed by the loss of HCl, reflects the presence of intact chlorine atom in **M26**.

*M27 ([M+H]* ^*+*^ *; m/z 455*.*1814)*: The metabolite **M27** at *m/z* 455.1814 ([M+H]^+^) with an elemental composition of C_6_H_16_N_2_O_5_PtS_2_ (2.66 ppm) was eluted at 7.6 min. The HRMS data suggests the lack of one chlorine atom and inclusion of hydroxy group in **M27** as compared to **M26**. This can be seen from the LC-MS/MS spectrum of protonated **M27** which displays an abundant ion at m/z 437.1877 (C_6_H_14_N_2_O_4_PtS_2_; 2.66 ppm) formed by the loss of H_2_O. This has been confirmed by the MS/MS of deuterated **M27** which showed the loss of D_2_O. Similarly to **M26**, the LC-MS/MS spectrum of protonated **M27** shows abundant product ions at m/z 333.1755 (C_3_H_8_NO_2_S), m/z 409.1454 (loss of HCOOH) and m/z 122.0275 (protonated cysteine) (Fig 13), represents the presence of strain chain methionine in its structure. Further, the formation an abundant product ion at m/z 411.1658 which involves the loss of CO_2_ and absence of the m/z 228.4812 ion (Pt^+^N_2_H_5_) (discussed in protonated **CP** and **M1-M17**), authenticates the presence of chelated cysteine ring in **M27**.

*M28 ([M+H]* ^*+*^ *; m/z 379*.*0851)*, *M29 ([M+H]* ^*+*^ *; m/z 494*.*5511)*, *M30 ([M+H]* ^*+*^ *; m/z 351*.*1822) and M31 ([M+H]* ^*+*^ *; m/z 438*.*4955)*: The metabolites **M28** at m/z 379.0851 (([M+H]^+^); C_5_H_18_N_3_O_2_PtS; 3.77 ppm), **M29** at m/z 494.5511 (([M+H]^+^); C_10_H_23_N_2_O_4_PtS_2_; -3.88 ppm), **M30** at m/z 351.1822 (([M+H]^+^); C_3_H_14_N_3_O_2_PtS; 1.88 ppm) and **M31** at m/z 438.4955 (([M+H]^+^); C_6_H_15_N_2_O_4_PtS_2_; -4.55 ppm) were eluted at 25.9, 10.6, 26.8 and 11.8 min, respectively. The LC-MS/MS spectra of protonated **M28** and **M30** display abundant product ions at m/z 335.1655 (C_4_H_18_N_3_PtS; 2.87 ppm) and m/z 307.1344 (C_2_H_14_N_3_PtS; 2.61 ppm) which were formed by the elimination of CO_2_ from the protonated molecular ion, clearly indicates the presence of chelated monomethionine and chelated monocysteine rings in **M28** and **M30**, respectively. It can be noted that the appearance of diagnostic product ion at m/z 228.4812 in the LC-MS/MS spectrum of protonated **M28** and **M30,** which is also a characteristic ion observed in the LC-MS/MS spectra of protonated **CP** and metabolites **M1-M17**, confirms the presence of Pt^+^N_2_H_5_ group. Based on these data, **M28** and **M30** were characterized to be chelated monomethionine and monocysteine CP metabolites, respectively.

Similarly, the LC-MS/MS spectra of protonated **M29** and **M31** display abundant product ions at m/z 450.4981 (C_9_H_23_N_2_O_2_PtS_2_; 4.11 ppm) & m/z 406.5011 (C_8_H_23_N_2_PtS_2_; 2.69 ppm) and m/z 394.5011 (C_5_H_15_N_2_O_2_PtS_2_; -4.07 ppm) & m/z 350.4968 (C_4_H_15_N_2_PtS_2_; -3.56 ppm), respectively, formed by the sequential elimination of one & two CO_2_ molecules, respectively, clearly indicates the presence of bismethionine and biscysteine chelated rings in **M29** and **M31**, respectively. Further, the absence of m/z 228.4812 ion in the LC-MS/MS spectra of **M29** and **M31,** which is diagnostic for the presence of Pt^+^N_2_H_5_ group (discussed in protonated **CP** and **M1-M17**), substantiates the presence of bismethionine and biscysteine rings in the structures of **M29** and **M31**, respectively. Based on these data, **M29** and **M31** were identified as bismethionine and biscysteine CP metabolites, respectively.

## Conclusions

A total of thirty one *in vivo* metabolites of CP formed in rat kidney tissue homogenate samples, have been identified and characterized by using liquid chromatography positive ion electrospray ionization high resolution tandem mass spectrometry (LC/ESI-HR-MS/MS). The structures of identified metabolites have been elucidated based on fragmentation pattern, accurate mass measurements combined with online HDX LC-MS/MS experiments. This study describes the utility of online deuterated experiments in the identification and structural characterization of drug metabolites. The dissociation of one of the chlorine atoms from CP results in a positive charge on the platinum that will attract the negatively charged sulfur present on the active thio group containing molecules. The results showed that CP undergoes a series of ligand exchange biotransformation reactions with water and active thio group containing nucleophiles like methionine, cysteine, acetylcysteine, glutathione and thioether to form various toxic metabolites. The major metabolites of CP are, mono aqua CP, monohydroxy CP, dihydroxy CP, hydroxy aqua CP, mono methionine CP, mono hydroxylated methionine CP, bismethionine CP, mono cysteine CP, mono hydroxylated cysteine CP, biscysteine CP, mono acetylcysteine CP, mono hydroxylated acetylcysteine CP, mono glutathione CP, mono hydroxylated glutathione CP, mono thioether CP, mono hydroxylated thioether CP. The newly identified metabolites have a scope to be assessed for their efficacy and toxicity. The structural identification of these metabolites might provide essential information for further pharmacological and clinical studies of CP, and may also be useful to develop various effective new anticancer agents. We further focus on the functions and toxicology studies of CP and its metabolites including nephrotoxicity in our future study. However, from the present it is difficult to comment on the toxic studies of CP and its metabolites in detail.
